# Supplementary material for: Trends in inequalities in avoidable hospitalisations across the COVID-19 pandemic: a cohort study of 23.5 million people in England
Source: BMJ Open. 2024 Jan 8;14(1):e077948. doi: 10.1136/bmjopen-2023-077948 (PMC10806625; doi:10.1136/bmjopen-2023-077948)
Supplement: Supplementary data [file bmjopen-2023-077948supp001.pdf]

Appendix

Table A: Missing data for covariates

Measure	Percent missing (%)
Index of Multiple Deprivation	1.68
Ethnicity	10.21
Government Office Region	0.05

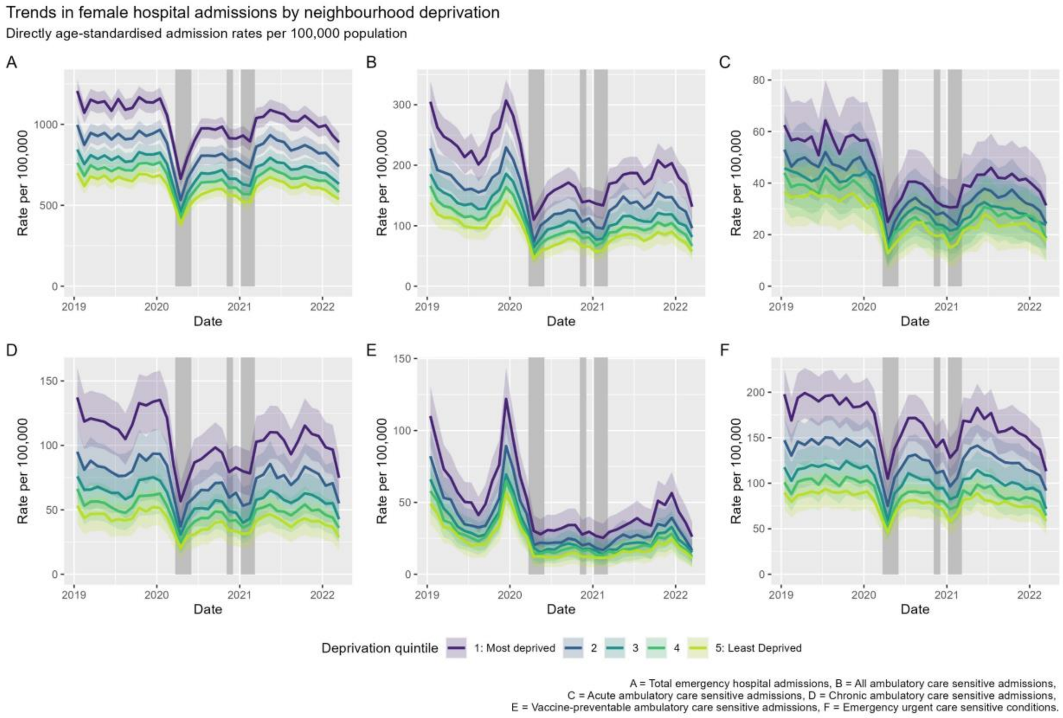

Figure A: Directly age-standardised admission rates for females by socioeconomic deprivation quintile. Note: shaded periods represent national lockdowns.

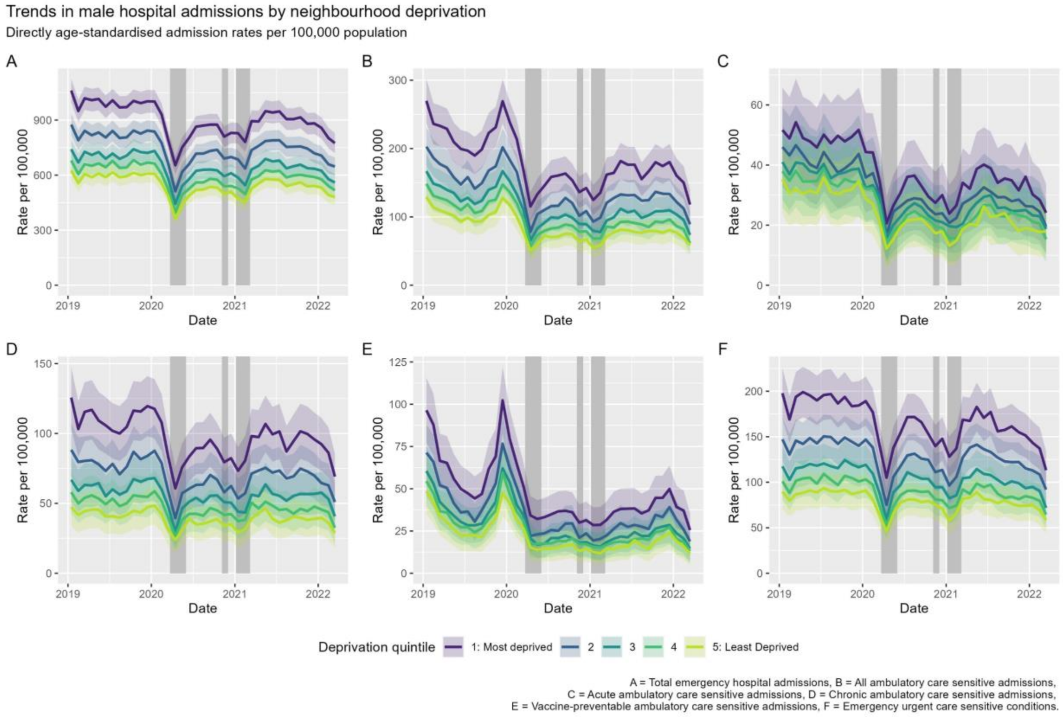

Figure B: Directly age-standardised admission rates for males by socioeconomic deprivation quintile. e: shaded periods represent national lockdowns.

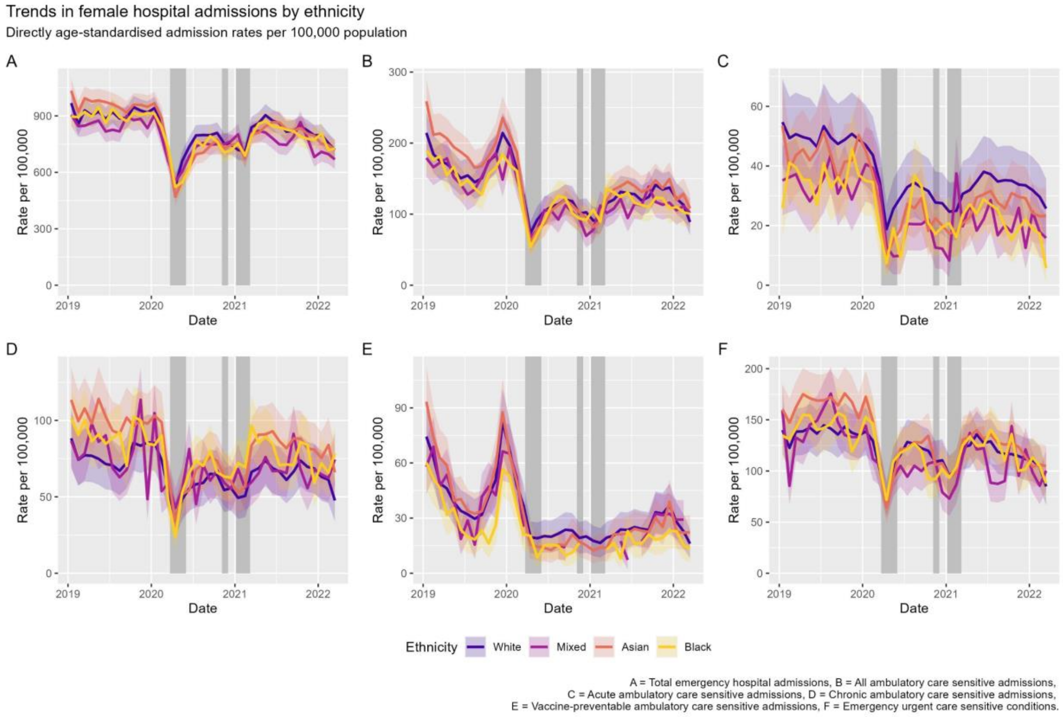

Figure C: Directly age-standardised admission rates for females by ethnicity. e: shaded periods represent national lockdowns.

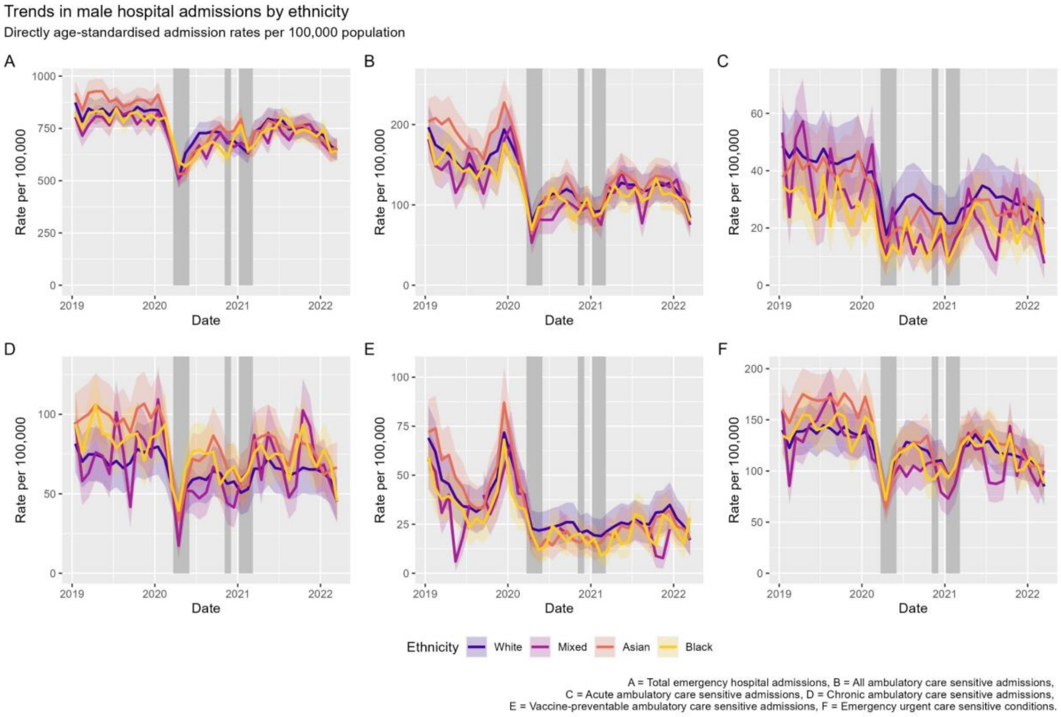

Figure D: Directly age-standardised admission rates for males by ethnicity. e: shaded periods represent national lockdowns.

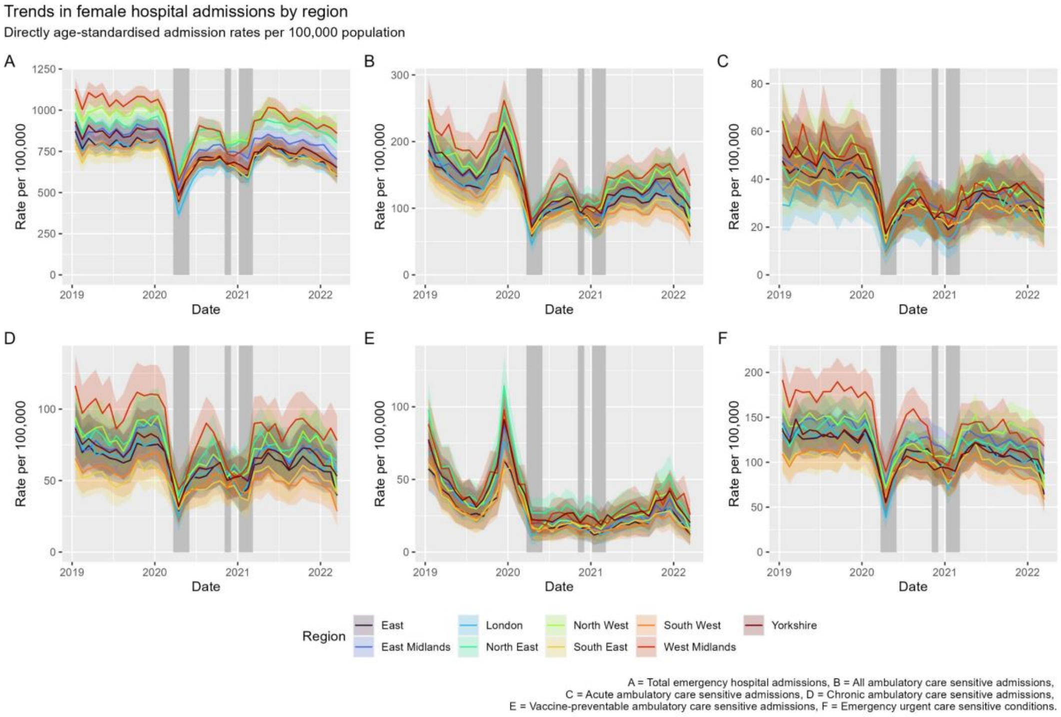

Figure E: Directly age-standardised admission rates for females by region. e: shaded periods represent national lockdowns.

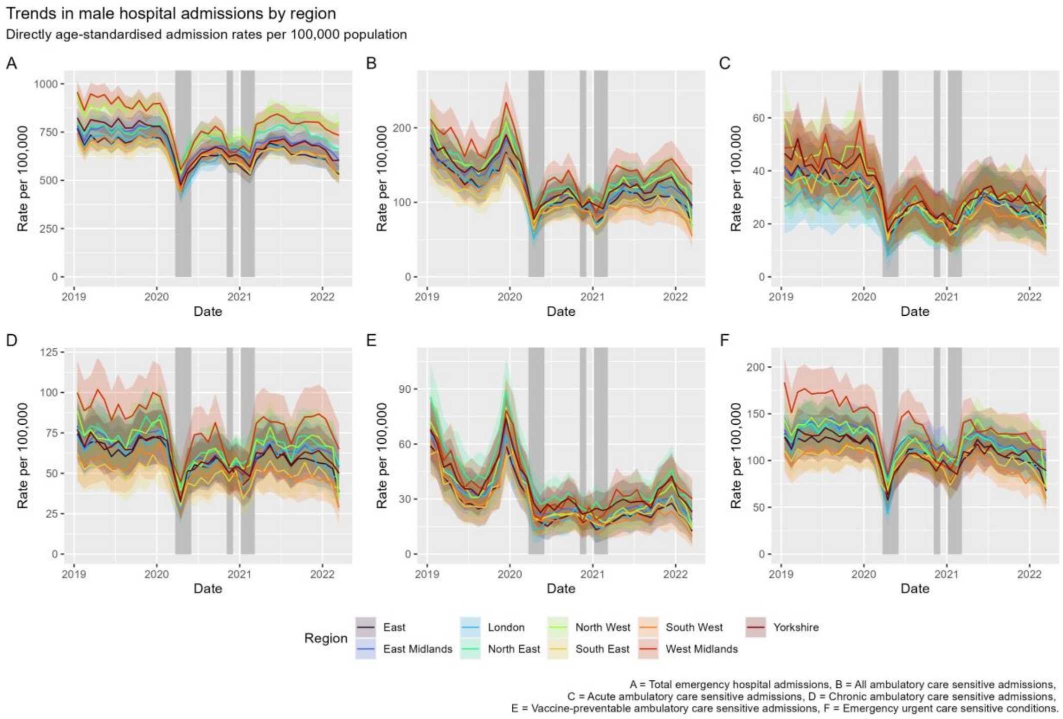

Figure F: Directly age-standardised admission rates for males by region. e: shaded periods represent national lockdowns.
