# Supplementary material for: Trends in inequalities in avoidable hospitalisations across the COVID-19 pandemic: a cohort study of 23.5 million people in England
Source: BMJ Open. 2024 Jan 8;14(1):e077948. doi: 10.1136/bmjopen-2023-077948 (PMC10806625; doi:10.1136/bmjopen-2023-077948)
Supplement: Author's manuscript [file bmjopen-2023-077948.draft_revisions.pdf]

BMJ Open is committed to open peer review. As part of this commitment we make the peer review history of every article we publish publicly available.

When an article is published we post the peer reviewers' comments and the authors' responses online. We also post the versions of the paper that were used during peer review. These are the versions that the peer review comments apply to.

The versions of the paper that follow are the versions that were submitted during the peer review process. They are not the versions of record or the final published versions. They should not be cited or distributed as the published version of this manuscript.

BMJ Open is an open access journal and the full, final, typeset and author-corrected version of record of the manuscript is available on our site with no access controls, subscription charges or pay-per-view fees (http://bmjopen.bmj.com).

If you have any questions on BMJ Open's open peer review process please email info.bmjopen@bmj.com

BMJ Open

Trends in inequalities in avoidable hospitalisations across the COVID-19 pandemic: A cohort study of 23.5 million people in England

Journal:	*BMJ Open*
Manuscript ID	bmjopen-2023-077948
Article Type:	Original research
Date Submitted by the Author:	19-Jul-2023
Complete List of Authors:	Green, Mark; University of Liverpool, Geography & Planning<br>McKee, Martin; European Centre on Health of Societ,<br>Massey, Jon; Oxford University, Nuffield Department of Primary Care Health Sciences<br>Mackenna, Brian; NHS England, Medicines and Diagnostics Policy Unit<br>Mehrkar, Amir; Oxford University, Nuffield Department of Primary Care Health Sciences<br>Bacon, Seb; University of Oxford, Nuffield Department of Primary Care Health Sciences<br>Macleod, John; University of Bristol<br>Sheikh, Aziz; University of Edinburgh, Division of Community Health Sciences<br>Shah, Syed Ahmar; The University of Edinburgh Usher Institute of Population Health Sciences and Informatics,<br>Katikireddi, Srinivasa; University of Glasgow, MRC/CSO Social & Public Health Sciences Unit<br>Consortium, The OpenSAFELY ; Oxford University<br>National Core Study Collaborative, The Longitudinal Health and Wellbeing; UCL
Keywords:	Quality in health care < HEALTH SERVICES ADMINISTRATION & MANAGEMENT, Rationing < HEALTH SERVICES ADMINISTRATION & MANAGEMENT, Health policy < HEALTH SERVICES ADMINISTRATION & MANAGEMENT, Public health < INFECTIOUS DISEASES, Population Dynamics, PUBLIC HEALTH

SCHOLARONE™
Manuscripts

I, the Submitting Author has the right to grant and does grant on behalf of all authors of the Work (as defined in the below author licence), an exclusive licence and/or a non-exclusive licence for contributions from authors who are: i) UK Crown employees; ii) where BMJ has agreed a CC-BY licence shall apply, and/or iii) in accordance with the terms applicable for US Federal Government officers or employees acting as part of their official duties; on a worldwide, perpetual, irrevocable, royalty-free basis to BMJ Publishing Group Ltd ("BMJ") its licensees and where the relevant Journal is co-owned by BMJ to the co-owners of the Journal, to publish the Work in this journal and any other BMJ products and to exploit all rights, as set out in our licence.

The Submitting Author accepts and understands that any supply made under these terms is made by BMJ to the Submitting Author unless you are acting as an employee on behalf of your employer or a postgraduate student of an affiliated institution which is paying any applicable article publishing charge ("APC") for Open Access articles. Where the Submitting Author wishes to make the Work available on an Open Access basis (and intends to pay the relevant APC), the terms of reuse of such Open Access shall be governed by a Creative Commons licence – details of these licences and which Creative Commons licence will apply to this Work are set out in our licence referred to above.

Other than as permitted in any relevant BMJ Author's Self Archiving Policies, I confirm this Work has not been accepted for publication elsewhere, is not being considered for publication elsewhere and does not duplicate material already published. I confirm all authors consent to publication of this Work and authorise the granting of this licence.

For peer review only

Trends in inequalities in avoidable hospitalisations across the COVID-19 pandemic: A cohort study of 23.5 million people in England

Mark A. Green[1,2], Martin McKee[3,4], Jon Massey[5,6], Brian MacKenna[5,7], Amir Mehrkar[5,6], Sebastian Bacon[5,6], John Macleod[8,9], Aziz Sheikh[10], Syed Ahmar Shah[10], The OpenSAFELY Consortium, The LH&W NCS Collaborative and Srinivasa Vittal Katikireddi[2].

[1] Geographic Data Science Lab, Department of Geography & Planning, University of Liverpool, Liverpool, UK.
[2] MRC/CSO Social and Public Health Sciences Unit, University of Glasgow, Glasgow, UK.
[3] Department of Health Services Research and Policy, London School of Hygiene and Tropical Medicine, London, UK.
[4] UCL Health Partners, London, UK.
[5] Bennett Institute for Applied Data Science, University of Oxford, Oxford, UK.
[6] Nuffield Department of Primary Care Health Sciences, University of Oxford, Oxford, UK.
[7] NHS England Medicines and Diagnostics Policy Unit, NHS England, London, UK.
[8] Population Health Sciences, University of Bristol, Bristol, UK.
[9] The National Institute for Health and Care Research Applied Research Collaboration West (NIHR ARC West) at University Hospitals Bristol and Weston NHS Foundation Trust, UK
[10] Usher Institute, University of Edinburgh, Edinburgh, UK.

Corresponding author
Mark Green. Address: Roxby Building, University of Liverpool, Liverpool, L69 7ZT. Email: mark.green@liverpool.ac.uk. Tel: +44 151 794 2854.

For peer review only

ABSTRACT

Objective: To determine whether periods of disruption were associated with increased 'avoidable' hospital admissions and wider social inequalities in England.

Design: Observational repeated cross-sectional study.

Setting: England (January 2019 to March 2022).

Participants: With the approval of NHS England we utilised individual-level electronic health records from OpenSAFELY, which covered ~40% of general practices in England (mean monthly population size 23.5 million people).

Primary and secondary outcome measures: We estimated crude and directly age-standardised rates for potentially preventable unplanned hospital admissions: ambulatory care sensitive conditions and urgent emergency sensitive conditions. We considered how trends in these outcomes varied by three measures of social and spatial inequality: neighbourhood socioeconomic deprivation, ethnicity, and geographical region.

Results: There were large declines in avoidable hospitalisations during the first national lockdown (March-May 2020). Trends increased post-lockdown but never reached 2019 levels. The exception to these trends was for vaccine-preventable ambulatory care sensitive admissions which remained low throughout 2020-2021. While trends were consistent by each measure of inequality, absolute levels of inequalities narrowed across levels of neighbourhood socioeconomic deprivation, Asian ethnicity (compared to White ethnicity), and geographical region (especially in northern regions).

Conclusions: We found no evidence that periods of healthcare disruption from the COVID-19 pandemic resulted in more avoidable hospitalisations. Falling avoidable hospital admissions has coincided with declining inequalities most strongly by level of deprivation, but also for Asian ethnic groups and northern regions of England.

Keywords

COVID-19; avoidable hospitalisations; electronic health records; inequality; health systems; disruption.

Strengths and weaknesses

- Utilises electronic health records for ~23.5M people which are representative of England.
- Examines how trends in avoidable hospitalisations vary across three measures of social and spatial inequality (deprivation, ethnic group and geographic region).
- Analyses are descriptive and unable to explain what factors were driving trends.
- It us uncertain whether avoidable hospitalisations are a good proxy measure for capturing the impacts of healthcare disruption.

For peer review only - http://bmjopen.bmj.com/site/about/guidelines.xhtml

INTRODUCTION

There has been widespread public, political, and media concern that the unprecedented disruption in accessing healthcare during the COVID-19 pandemic would lead to an increase in hospital admissions as a result of people not receiving the care that they needed. The levels of disruption have been widely described. Visits to general practice fell markedly (1), although this was later compensated for by a rise in online consultations (2). There were three million fewer elective treatment pathways in England in 2020 than compared to 2019 (3). Cancer screening programmes, non-essential surgeries and diagnostic procedures were postponed or cancelled (1,4). Waiting lists have continued to get longer, resulting in delayed access to care for new treatments (5,6). High levels of staff illness from both acute COVID-19 and, increasingly, Long COVID, have caused significant disruption that continues to affect the level of care delivered (7). These trends have been observed not only in England, but also many countries internationally (8,9). There has been little empirical evaluation into whether periods of disruption actually translated into more hospital admissions. We are only aware of one study which demonstrated that people who self-reported that they were disrupted in accessing healthcare during COVID-19 had higher odds of a hospital admission for a potentially preventable condition in the first two and a half years of the pandemic (10).

Identifying the impacts of healthcare disruption is difficult. The journey from onset of illness to accessing care is complex and obstacles can arise at many points across the health system (11). To simplify this issue, we borrowed a key concept from the health systems literature, 'avoidable hospital admissions', which is considered a proxy measure for health system performance (12). Avoidable hospitalisations are emergency (unplanned) hospital admissions that could have potentially been prevented if individuals had received timely care within the community (and therefore may be susceptible to disruptions to care) (12,13). Reducing avoidable hospital admissions is a priority for the NHS because they are often costly and disrupt elective care (14). Significant disruption to healthcare access in the community associated with uncontrolled infection and non-pharmaceutical interventions implemented in response (e.g., people unable to see their GP or postponement to treatments) may have resulted in more avoidable hospitalisations where individuals were not able to receive the care they needed.

Research has showed that avoidable hospitalisations in many countries fell in 2020 during the first COVID-19 waves and did not return to pre-pandemic levels thereafter (15–18). Most of this research, however, stops at the end of 2020 and there is less understanding of how trends have continued since. This ignores a critical period in the response and recovery from the pandemic. 2021-2022 saw significant disruption to health systems due to COVID-19 (19). It is also plausible that the impacts of disrupted access to healthcare are not immediate and may only be observable in the medium-term (e.g., delayed diagnosis or treatment leading to disease progression that results in an avoidable hospitalisation a year down the line). Much of this research has been undertaken using small datasets for specific geographic contexts (e.g. single cities or states) which may not be generalisable to the national level. Few studies have examined how trends in avoidable hospitalisations have varied across measures of social inequalities. This is pertinent since the COVID-19 pandemic shined a light on the fractures within English society (20), with people of lower socioeconomic position, minoritised ethnic groups, and those living in parts of northern England disproportionally affected by COVID-19 (21–23). These same communities have not

For peer review only - http://bmjopen.bmj.com/site/about/guidelines.xhtml

just only been shown to report greater experiences of healthcare disruption (24), but they are also those which had higher rates of avoidable hospitalisations pre-pandemic too (12,25–27). We hypothesise that the COVID-19 pandemic may have widened social inequalities in avoidable hospitalisations.

The aim of our study is to determine whether periods of healthcare disruption were associated with increased ‘avoidable’ hospital admissions and wider social inequalities in England.

METHODS

Data

The primary data source used for the analysis was OpenSAFELY-TPP. OpenSAFELY is an open-source secure health data analysis platform providing access to primary care records, linked to secondary care and mortality records, held by the two largest electronic health record providers for NHS England – Egton Medical Information Systems (EMIS) and The Phoenix Partnership (TPP) which cover ~58 million registered patients. In this study, we had access to data from TPP (The Phoenix Partnership) which covers ~40% of general practices in England. The data are broadly representative of England by age, sex, ethnicity, small area socioeconomic deprivation and cause of death (28).

Data were accessed on 20th May 2022. The study period for our analysis was 1st January 2019 to 31st March 2022. While 2019 only provides one year’s worth of pre-pandemic data, trends prior to 2019 were relatively flat (29). We discounted all data in April and May 2022 to minimise under-counting of events due to possible delays in reporting of clinical information relating to admissions. Total counts per month were rounded to their nearest 5 to minimise patient disclosure risks where low counts were evident. All analytical scripts, processed data and outputs are openly available at https://github.com/opensafely/avoidable_hospitalisations_trends.

Outcomes

Avoidable hospitalisations were defined using five measures commonly used by NHS England (12). Each were defined using ICD-10 codes (codelists openly available at (30)), primary diagnosis (diagnostic position 1) and for emergency (unplanned) admissions only. All ambulatory care sensitive conditions were selected as an overall proxy for avoidable hospitalisations. Ambulatory care sensitive conditions were defined as conditions that can be treated effectively in the community and should not therefore require hospital admission (12,13,29). We further disaggregated all ambulatory care sensitive conditions into three measures representing the type of condition: (i) acute conditions (e.g., cellulitis, dental caries, rickets, gastric ulcer), (ii) chronic conditions (e.g., hypertension, angina, asthma), (iii) vaccine-preventable conditions (e.g., mumps, measles, influenza). We did not include COVID-19 admissions as avoidable or vaccine-preventable conditions as we wanted to examine the indirect impacts of the pandemic (as well as ensuring a consistent set of conditions pre-pandemic). We also included emergency urgent care sensitive conditions as an alternative measure of avoidable hospitalisations. These are acute exacerbations of urgent conditions that will result in hospital admission but that the NHS should be able to treat within the community to minimise the need for hospital care (12,13,29). Finally, we

For peer review only - http://bmjopen.bmj.com/site/about/guidelines.xhtml

also included a measure of all emergency hospital admissions to provide context for our measures.

Measures of social inequality

We considered how trends in our outcome variables varied by three measures of social and spatial inequality that have been widely reported to have been associated with unequal COVID-19 outcomes (20,23). Neighbourhood socioeconomic deprivation was measured using the Index of Multiple Deprivation 2019 (IMD) (31). Individuals were matched to Lower Super Output Area by their home residence in each month and we calculated the quintile of deprivation rank. Ethnicity was recorded in the electronic health record and we consider four groups (White or White British, Black or Black British, Asian or Asian British, and Mixed ethnicity). We did not undertake further disaggregation because of issues with small numbers. We excluded individuals with 'Other' ethnicity since the group is extremely heterogeneous, limiting our scope to draw conclusions (they represented 2.2% of the entire sample). Patients were allocated to seven Government Office Regions, based on home residence, to capture regional differences. Missing data for covariates are presented in Appendix Table A.

Statistical analyses

We estimated summary statistics for each outcome measure using aggregated counts from individual records. Where aggregated statistics were calculated, we used crude rates to measure monthly hospital admission rates (population was defined as total number of people alive on the 1st of each month within the OpenSAFELY-TPP data). Where we stratified measures by socio-demographic indicators, we estimated directly age-standardised rates to adjust for the different age structures of each group that may confound trends. All measures were stratified by sex (female or male). We visualised rates and presented descriptive statistics to investigate trends. To quantify and measure the extent of how trends in inequalities have changed over time, we used a difference-in-difference regression model. Here we fit a linear regression model using the monthly values as inputs. The directly age-standardised admission rate was used as our outcome, with a binary predictor variable for if the month was pre- or post-pandemic (defined here as March 2020 onwards), a categorical variable for the groups in a socio-demographic measure of inequality (separate models for each measure), and an interaction effect between the binary and categorical variables. We focus on the interaction effect here since it tells us how the trend changed overall. We excluded all missing data from analyses.

Patient and Public Involvement

There was no public engagement in the design or completion of this project.

RESULTS

Overall population-level trends

Table 1 presents key summary statistics for our data. During the study period, there were 6 645 550 emergency hospital admissions, with a monthly average of 170 399. There were 1 129 770 ambulatory care sensitive hospital admissions (17% of all emergency admissions), chronic ambulatory care hospital admissions being most common. There 1 031 205 emergency urgent care hospital admissions (16% of all emergency admissions).

For peer review only - http://bmjopen.bmj.com/site/about/guidelines.xhtml

Table 1: Frequency counts for outcome measures (1st January 2019 to 31st March 2022).

Measure	Total over study period	Mean per month
Emergency admissions	6,645,550	170,399
All ambulatory care admissions	1,129,770	28,968
Acute ambulatory care admissions	292,630	7,503
Chronic ambulatory care admissions	563,740	14,455
Vaccine-preventable ambulatory care admissions	282,630	7,247
Emergency urgent care sensitive	1,031,205	26,441
Population	918,302,035	23,546,206

Figure 1 presents crude admission rates by sex over the study period for each of our outcome measures. Trends in emergency hospital admissions were stable and consistent throughout 2019 (Figure 1A). In 2020, there were sudden large falls in emergency admissions coinciding with the first national lockdown in England (e.g., in April 2020 rates were 44% lower (females) and 39% lower (males) than compared to rates in January 2020). Emergency admission rates then increased quickly in the period following the national lockdown, although increases did not reach the same levels as in 2019. Rates fell once more at the end of 2020 (again coinciding with national lockdowns), subsequently recovering quickly and remaining at a level higher in 2021 than in 2020, but still less than in 2019.

Patterns of avoidable hospitalisations largely follow similar trends to emergency hospital admissions. There were large falls in all, acute, and chronic ambulatory sensitive hospital admissions and emergency urgent sensitive admissions between March and May 2020. These falls were then followed by large rises, albeit not to 2019 levels, before subsequent falls during the second and third national lockdowns (with large rises following). Vaccine-preventable ambulatory hospital admissions were the only measure that did not follow this trend. There were distinct peaks in winter 2019 but, after December, rates fell sharply and then continued at a low level throughout all of 2020. Rates then began to rise throughout 2021 (although only to the lowest points of 2019), peaking in December 2021, before falling thereafter.

[Figure 1 here]

Figure 1: Crude hospital admission rates (per 100,000 population) by sex for measures of avoidable hospitalisations. Note: shaded periods represent national lockdowns.

Trends by deprivation

We next examined whether trends in our measures varied by deprivation. Appendix Figures A and B present trends for females and males respectively, although there were minimal differences by sex so we described the findings together. A distinct social gradient was evident for all outcomes, with the highest rates in the most deprived quintile and lowest in the least deprived quintile. Trends over the study period largely followed those reported at the population level and were consistent across deprivation quintiles.

For peer review only - http://bmjopen.bmj.com/site/about/guidelines.xhtml

Figure 2 presents the interaction effects from our difference-in-difference models. The associations are negative which suggest that inequalities by deprivation narrowed over the study period. Only for the most deprived quintile did the 95% confidence intervals not cross a value of 0 for all outcomes. The extent of this narrowing of inequalities was not consistent throughout the study period. The range between most and least deprived quintiles was smallest during the first national lockdown. For example, the range for females was 65 per 100,000 in April 2020 compared to 156 per 100,000 in January 2020. The range of values then increased following the lockdown but always remained smaller than what was observed in 2019. This would support the notion that absolute levels of social inequalities by deprivation have narrowed. However, relative differences remained similar (e.g., 2.4 times higher in April 2020 compared to 2.3 times higher in January 2020 for females) suggesting that inequalities were still important determinants.

[Figure 2]

Figure 2: Estimated interaction effects (with 95% confidence intervals) between start of the pandemic and deprivation quintile from a series of difference-in-difference regression models for six outcome variables. Note: EUCS = Emergency Urgent Care Sensitive. Ambulatory = ambulatory care sensitive conditions. Least deprived quintile was selected as the reference group.

Trends by ethnicity

Appendix Figures C and D present trends by ethnic group and sex. The general trends for each outcome match those described in earlier sections, with the falls in hospital admissions during key COVID-19 waves consistent across all ethnic groups. While there is some ordering of rates by ethnic group, the ordering itself is not consistent and confidence intervals often overlap, suggesting that any ordering is not meaningful. There were no clear observable differences between groups during the main waves/lockdowns.

When estimating how trends changed overall (Figure 3), we did not find evidence of significantly different trends for mixed and black ethnic groups in comparison to white ethnicity. For Asian ethnicity, we find evidence of negative associations which suggests that trends in avoidable hospitalisations declined at a greater rate than compared to the white ethnic group over the period.

[Figure 3 here]

Figure 3: Estimated interaction effects (with 95% confidence intervals) between start of the pandemic and ethnic group from a series of difference-in-difference regression models for six outcome variables. Note: EUCS = Emergency Urgent Care Sensitive. Ambulatory = ambulatory care sensitive conditions. White ethnicity was selected as the reference group.

Trends by region

Appendix Figures E and F present trends by geographical region for England. Trends largely follow those described previously, with falls in admissions across all outcomes during national lockdowns consistent across all regions. There were noticeable inequalities between regions. Rates in the West Midlands were consistently highest for most outcomes

For peer review only - http://bmjopen.bmj.com/site/about/guidelines.xhtml

(other than vaccine-preventable admissions), followed by North West and North East. Admission rates in the South East and South West were consistently lowest across all periods and for all outcomes. London is the exception, lying in the middle for most outcomes. However, during the first national lockdown (and to a lesser extent at the end of 2020), falling rates take London to the lowest values.

When estimating the overall change in trends, we found inconsistent patterns (Figure 4). Yorkshire and West Midlands had the greatest number of estimates that were statistically significant. Northern regions also saw negative associations in ambulatory care sensitive conditions for females, but not males (in comparison to the South East).

[Figure 4 here]

Figure 4: Estimated interaction effects (with 95% confidence intervals) between start of the pandemic and geographic office region from a series of difference-in-difference regression models for six outcome variables. Note: EUCS = Emergency Urgent Care Sensitive. Ambulatory = ambulatory care sensitive conditions. South East region was selected as the reference group.

DISCUSSION

Key results

Using electronic health records for an average of 23.M people per month, we demonstrate that the COVID-19 pandemic led to a step-change in 'avoidable' hospital admissions. All measures of avoidable hospitalisations we analysed declined during the first national lockdown. While rates increased subsequently (other than in periods of national lockdowns), avoidable hospitalisations have remained lower than 2019 levels. Vaccine-preventable ambulatory care sensitive conditions have remained low for 2020 and 2021, and did not follow trends for other measures of avoidable hospitalisations. Avoidable hospitalisations were highest in the most deprived areas, Yorkshire and West Midlands, and inconsistent across ethnic groups. We find evidence of narrowing absolute levels of inequalities by deprivation, ethnicity, and geographical region. The largest falls in inequalities occurred during the first national lockdown and have remained lower thereafter.

Interpretation

Our study is one of the largest to investigate how avoidable hospitalisations changed before and during the COVID-19 pandemic. We find large falls in avoidable hospitalisations during periods of national lockdowns. During periods when society opened up, rates increased but never to 2019 levels suggesting a gradual recovery in population-level trends in avoidable hospitalisations. These findings follow similar trends observed in other countries despite their differences in health systems, socio-cultural contexts and experiences of COVID-19 (15–18). We extend on this work through analysing a longer time period and through detailed disaggregated by markers of socioeconomic inequality. Our results also follow observations for other metrics including falls in non-COVID-19 mortality (32,33).

While one interpretation might therefore be that the impacts of healthcare disruption were minimal, we should not forget that healthcare disruption is part of the explanation for the

For peer review only - http://bmjopen.bmj.com/site/about/guidelines.xhtml

trends we observe. During the first national lockdown, the NHS cancelled or postponed or many patients planning to be admitted to free capacity for patients with COVID-19 (3,4,6). Individuals may also have avoided going to hospital if at all possible for fear of being exposed to SARS-CoV-2 (34). Such disruption may explain why we saw the largest decreases in rates during the first lockdown; the NHS was trying to avoid admitting people to hospital unless events were life threatening or required urgent care. More people dying at home may reflect this process (35), although the magnitude of increase is unlikely fully to explain our results especially the narrowing of inequalities. Similarly, COVID-19 and its legacy may have resulted in a permanent shock to the health system that has reduced the number of admitted care the NHS can provide. This would follow evidence elsewhere that the NHS is under “record pressures” (36). The extent of declines in trends is likely to reflect significant changes in patient behaviour (e.g., fear of being exposed to SARS-CoV-2 in healthcare settings, not wanting to burden services in a crisis) and access to health systems (e.g., physicians deciding not to refer patients to hospital, changes in how admissions are made, prioritisation of community care). Unpicking the exact drivers is complex given the numerous interacting factors that may have produced this drop in avoidable hospitalisations.

Vaccine-preventable ambulatory care sensitive hospital admissions had markedly different trends to the other types of avoidable hospitalisations. Low levels of vaccine-preventable ambulatory care hospital admissions in 2020-2021 reflects the low level of influenza (37). Influenza normally accounts for a large proportion of these hospital admissions but, as with all respiratory infections, of its transmission is reduced by the non-pharmaceutical interventions adopted for SARS-CoV-2 (38,39). Reductions in other viral and respiratory conditions such as asthma exacerbations also follow these trends (33). However, now that these measures have been relaxed, there is a need to prepare for co-occurring waves of SARS-CoV-2 and influenza (40).

The impacts of the COVID-19 pandemic have in many respects been highly unequal (20). While our results provide evidence on the unequal distribution of avoidable hospitalisations across social and spatial characteristics, the changing trends in inequalities are not always so obvious, consistent and often nuanced in their interpretation. Contrary to our hypothesis, we find evidence that absolute levels of inequalities in avoidable hospitalisations across deprivation, ethnicity and region narrowed. Counterintuitively for deprivation, the narrowing of inequalities occurred during the first national lockdown, suggesting that the period of greatest disruption in the access to healthcare did not disproportionally impact people in these areas. We cannot discount entirely the possibility that this is a consequence of fewer hospital admissions during these periods. It may be that individuals who would have normally been captured in avoidable hospitalisations had experienced COVID-19, been hospitalised and died as a result instead (especially given the disproportionate distribution of unplanned hospitalisation and COVID-19 outcomes by our measures of inequality). However, we did not find evidence that inequalities returned back to 2019 levels during periods of low disruption or outside of COVID-19 waves which would suggest the driver is different.

Although inequalities narrowed, there were still differences between regions, ethnic groups and levels of deprivation reminding us that inequalities remain important. More deprived

For peer review only - http://bmjopen.bmj.com/site/about/guidelines.xhtml

areas and northern regions had consistently higher avoidable hospitalisations throughout the whole study period. There was a lack of literature on ethnic inequalities in avoidable hospitalisations pre-pandemic (12), and our work contributes to showing how intricate they can be when investigating trends. Trends between Black and Mixed ethnic groups to White ethnicity were not different, which is different to experiences in the USA (16). Our findings reiterate the need for policy makers to focus on tackling the root structural causes of health. Health is socially determined (20). We need to better understand how and why the social determinants of health influence the risk of avoidable hospitalisations to identify areas we can intervene. These need to include both social (e.g., people in more deprived areas working multiple jobs which don't have time to see their GP and end up using more flexible emergency care instead) and spatial factors (e.g., are there cultural or infrastructure reasons that explain why northern regions perform poorly). While we do not find clear and consistent inequalities for ethnicity, this should not detract from the structural injustices that minoritised ethnic groups face which has implications for their health (21).

Limitations

Our analyses are descriptive and are unable to identify the reasons behind what the trends we observe. The findings may be subject to ecological fallacy as we had no measure of individual experience of healthcare disruption. However, the magnitude of changes are clear enough to suggest that they are real at least at the population level. While our electronic health records provide large representative data on patients (28), they lack the detail about the contexts of individual's lives that can be found in other data types (e.g., longitudinal surveys).

By using a proxy measure of health system performance, we are also unable to ascertain the extent that our findings do reflect actual disruption to healthcare. Its impacts will be complex, occurring over the short- and long-term. It may be that our study is too early to detect the full impact of this disruption and it will be necessary to continually monitor trends in our outcomes. Additionally, there is some debate in the literature over how valid avoidable hospitalisations are as a proxy for health system performance (12). Although our measures are used by NHS England for measuring health system performance (12), other COVID-19 related outcomes have witnessed widening social inequalities which are different to our findings (1,20,21). Regional and hospital differences in reporting patterns may also exist. Validating our chosen outcomes is necessary for understanding the importance of our findings.

We use coarse groupings for each of our measures of inequality. For example, we group individuals by broad ethnic groups that hide the diversity within each group (e.g., Asian constitutes communities from very different backgrounds and heritages). This was partly to minimise statistical disclosure issues, due to the lower number of events per month in some groups. Future research should utilise finer groupings that can better describe the extent of inequalities, ideally recognising the important of intersectionality, and help identify particular populations at greater risk of avoidable hospitalisations. Similarly, moving beyond describing geographical inequalities by region to identify how trends vary across smaller places can help to present more precise patterns and help to identify potential drivers.

For peer review only - http://bmjopen.bmj.com/site/about/guidelines.xhtml

CONCLUSIONS

The COVID-19 pandemic has affected avoidable hospitalisations in unexpected and potentially surprising ways. Worries that the pandemic would see rising hospital admissions due to disruptions in accessing care do not appear to have materialised, at least so far. While social and spatial inequalities narrowed during the pandemic, recent widening regional inequalities present cause for concern and present the case for renewed focus among narratives of 'building back better' and 'levelling up'.

REFERENCES

1 Mansfield KE, Mathur R, Tazare J, *et al.* Indirect acute effects of the COVID-19 pandemic on physical and mental health in the UK: a population-based study. *Lancet Digit Heal* 2021; **3**: e217–30.

2 Green MA, McKee M, Katikireddi SV. Remote general practitioner consultations during COVID-19. *Lancet Digit Heal* 2022; **4**: e7.

3 Warner M, Burn S, Stoye G, Aylin PP, Bottle A, Propper C. Socioeconomic deprivation and ethnicity inequalities in disruption to NHS hospital admissions during the COVID-19 pandemic: a national observational study. *BMJ Qual Saf* 2022; **31**: 590–8.

4 Maringe C, Spicer J, Morris M, *et al.* The impact of the COVID-19 pandemic on cancer deaths due to delays in diagnosis in England, UK: a national, population-based, modelling study. *Lancet Oncol* 2020; **21**: 1023–34.

5 BMA. NHS backlog data analysis 2022. 2022. https://www.bma.org.uk/advice-and-support/nhs-delivery-and-workforce/pressures/nhs-backlog-data-analysis (accessed July 15, 2022).

6 Gardner T, Fraser C. Longer waits, missing patients and catching up. 2021. https://www.health.org.uk/news-and-comment/charts-and-infographics/how-is-elective-care-coping-with-the-continuing-impact-of-covid-19

7 McKee M, Hiam L. Britain's not working. BMJ. 2022; **378**: o1883.

8 Bambra C, Riordan R, Ford J, Matthews F. The COVID-19 pandemic and health inequalities. *J Epidemiol Community Heal* 2020; **74**: 964–8.

9 Mathur R, Rentsch CT, Morton CE, *et al.* Ethnic differences in SARS-CoV-2 infection and COVID-19-related hospitalisation, intensive care unit admission, and death in 17 million adults in England: an observational cohort study using the OpenSAFELY platform. *Lancet* 2021; **397**: 1711–24.

10 Griffith GJ, Owen G, Manley D, Howe LD, Davey Smith G. Continuing inequalities in COVID-19 mortality in England and Wales, and the changing importance of regional, over local, deprivation. *Health Place* 2022; **76**: 102848.

11 NHS England. The NHS Long Term Plan. 2019. https://www.longtermplan.nhs.uk/

12 UK Government. Final report on progress to address COVID-19 health inequalities. 2021. https://www.gov.uk/government/publications/final-report-on-progress-to-address-covid-19-health-inequalities.

13 Tudor-Hart J. The inverse care law. *Lancet* 1971; **1**: 405–12.

14 Seguin ML, Rangnekar A, Renedo A, Palafox B, McKee M, Balabanova D. Systematic review of frameworks used to conceptualise health pathways of individuals diagnosed with cardiovascular diseases. *BMJ Glob Heal* 2020; **5**: e002464.

15 O'Cathain A, Knowles E, Turner J, *et al.* Explaining variation in emergency admissions: a mixed methods study of emergency and urgent care systems. *Heal Serv Deliv Res* 2014; **2**: 48.

For peer review only - http://bmjopen.bmj.com/site/about/guidelines.xhtml

16 Coleman P, Nicholl J. Consensus methods to identify a set of potential performance indicators for systems of emergency and urgent care. *J Health Serv Res Policy* 2010; **15 Suppl 2**: 12–8.

17 Andrews C, Schultze A, Curtis H, *et al.* OpenSAFELY: Representativeness of electronic health record platform OpenSAFELY-TPP data compared to the population of England. *Wellcome open Res* 2022; **7**: 191.

18 Nuffield Trust. Potentially preventable emergency admissions. 2022. https://www.nuffieldtrust.org.uk/resource/potentially-preventable-emergency-hospital-admissions#:~:text=In%20February%202022%2C%20the%20biggest,data%20section%20of%20this%20page.

19 Green MA. Open Codelists. 2022. https://www.opencodelists.org/users/mgreen/

20 Ministry of Housing Communities and Local Government. The English Indices of Deprivation 2019. 2019. https://www.gov.uk/government/statistics/english-indices-of-deprivation-2019 .

21 Mackenbach JP, Kunst AE. Measuring the magnitude of socio-economic inequalities in health: an overview of available measures illustrated with two examples from Europe. *Soc Sci Med* 1997; **44**: 757–71.

22 Tallack C, Krelle H. What has happened to non-COVID mortality during the pandemic? 2021. https://www.health.org.uk/publications/long-reads/what-has-happened-to-non-covid-mortality-during-the-pandemic

23 Shah SA, Brophy S, Kennedy J, *et al.* Impact of first UK COVID-19 lockdown on hospital admissions: Interrupted time series study of 32 million people. *eClinicalMedicine* 2022; **49**.

24 Watt T, Sullivan R, Aggarwal A. Primary care and cancer: an analysis of the impact and inequalities of the COVID-19 pandemic on patient pathways. *BMJ Open* 2022; **12**: e059374.

25 ONS. Deaths at home increased by a third in 2020, while deaths in hospitals fell except for COVID-19. 2021. https://www.ons.gov.uk/peoplepopulationandcommunity/birthsdeathsandmarriages/deaths/articles/deathsathomeincreasedbyathirdin2020whiledeathsinhospitalsfellexceptforcovid19/2021-05-07

26 Rocks S. Why is the NHS really under ‘record pressure’? 2022. https://www.health.org.uk/news-and-comment/charts-and-infographics/do-we-really-understand-why-the-nhs-is-under-record-pressure

27 UK Health Security Agency. Surveillance of influenza and other seasonal respiratory viruses in winter 2021 to 2022. 2022. https://www.gov.uk/government/statistics/annual-flu-reports/surveillance-of-influenza-and-other-seasonal-respiratory-viruses-in-winter-2021-to-2022

28 Huang QS, Wood T, Jelley L, *et al.* Impact of the COVID-19 nonpharmaceutical interventions on influenza and other respiratory viral infections in New Zealand. *Nat Commun* 2021; **12**: 1001.

29 Davis WW, Mott JA, Olsen SJ. The role of non-pharmaceutical interventions on influenza circulation during the COVID-19 pandemic in nine tropical Asian countries. *Influenza Other Respi Viruses* 2022; **16**: 568–76.

30 Nazareth J, Pan D, Martin CA, *et al.* Is the UK prepared for seasonal influenza in 2022-23 and beyond? *Lancet Infect Dis* 2022.

For peer review only - http://bmjopen.bmj.com/site/about/guidelines.xhtml

For peer review only

Competing interests
None declared.

Acknowledgements
We are very grateful for all the support received from the TPP Technical Operations team throughout this work, and for generous assistance from the information governance and database teams at NHS England and the NHS England Transformation Directorate. Information about data sharing, data access and information governance can be found at the end of this document. This work was funded as part of the National Core Studies Longitudinal Health & Wellbeing programme (MC_PC_20030, MC_PC_20059). MAG acknowledges funding for this research from UK Medical Research Council (MR/W021242/1). SVK acknowledges funding from a NRS Senior Clinical Fellowship (SCAF/15/02), the Medical Research Council (MC_UU_00022/2) and the Scottish Government Chief Scientist Office (SPHSU17). JM is partly funded by the National Institute for Health and Care Research Applied Research Collaboration West (NIHR ARC West). The full statement, listing the names of all relevant NCS Consortium staff, can be found here: https://www.ucl.ac.uk/covid-19-longitudinal-health-wellbeing/convalescence-study-collaborative.

Author contributions
MAG, MM and SVS came up with the idea for the study. MAG, MM and SVS designed the study, with input from AS, JM and SAS. JM, BM, AM and SB were involved in data access and collection. MAG and JM manipulated data into an analysis ready format. MAG led on data analyses, supported by SVS. MAG, MM and SVS wrote the initial draft of the paper, with all other authors revising the paper.

Data sharing statement
All data were linked, stored and analysed securely within the OpenSAFELY platform: https://opensafely.org/. Data include pseudonymised data such as coded diagnoses, medications and physiological parameters. No free text data are included. All code is shared openly for review and re-use under MIT open license on https://github.com/opensafely/avoidable_hospitalisations_trends. Detailed pseudonymised patient data is potentially re-identifiable and therefore not shared. Primary care records managed by the GP software provider, TPP, were linked to Hospital Episode Statistics through OpenSAFELY.

Information governance
NHS England is the data controller for OpenSAFELY-TPP and OpenSAFELY-EMIS; EMIS and TPP are the data processors; all study authors using OpenSAFELY have the approval of NHS England. This implementation of OpenSAFELY is hosted within the EMIS and TPP environments which are accredited to the ISO 27001 information security standard and are NHS IG Toolkit compliant.[1]

Patient data has been pseudonymised for analysis and linkage using industry standard cryptographic hashing techniques; all pseudonymised datasets transmitted for linkage onto OpenSAFELY are encrypted; access to the platform is via a virtual private network (VPN)

For peer review only - http://bmjopen.bmj.com/site/about/guidelines.xhtml

connection, restricted to a small group of researchers; the researchers hold contracts with NHS England and only access the platform to initiate database queries and statistical models; all database activity is logged; only aggregate statistical outputs leave the platform environment following best practice for anonymisation of results such as statistical disclosure control for low cell counts.[2]

The OpenSAFELY research platform adheres to the obligations of the UK General Data Protection Regulation (GDPR) and the Data Protection Act 2018. In March 2020, the Secretary of State for Health and Social Care used powers under the UK Health Service (Control of Patient Information) Regulations 2002 (COPI) to require organisations to process confidential patient information for the purposes of protecting public health, providing healthcare services to the public and monitoring and managing the COVID-19 outbreak and incidents of exposure; this sets aside the requirement for patient consent.[3] This was extended in July 2022 for the NHS England OpenSAFELY COVID-19 research platform.[4] In some cases of data sharing, the common law duty of confidence is met using, for example, patient consent or support from the Health Research Authority Confidentiality Advisory Group.[5]

Taken together, these provide the legal bases to link patient datasets on the OpenSAFELY platform. GP practices, from which the primary care data are obtained, are required to share relevant health information to support the public health response to the pandemic, and have been informed of the OpenSAFELY analytics platform.

This study was classified as service evaluation. This study was supported by Professor Martin McKee (Honorary Consultant at UCLH NHS Foundation Trust) as senior sponsor, and approved by the University of Liverpool's Research Ethics Board [reference 10634].

References:

1 Data Security and Protection Toolkit - NHS Digital. NHS Digital. https://digital.nhs.uk/data-and-information/looking-after-information/data-security-and-information-governance/data-security-and-protection-toolkit (accessed 30 Apr 2020).

2 ISB1523: Anonymisation Standard for Publishing Health and Social Care Data - NHS Digital. NHS Digital. https://digital.nhs.uk/data-and-information/information-standards/information-standards-and-data-collections-including-extractions/publications-and-notifications/standards-and-collections/isb1523-anonymisation-standard-for-publishing-health-and-social-care-data (accessed 30 Apr 2020).

3 Secretary of State for Health and Social Care - UK Government. Coronavirus (COVID-19): notification to organisations to share information. 2020. https://web.archive.org/web/20200421171727/https://www.gov.uk/government/publications/coronavirus-covid-19-notification-of-data-controllers-to-share-information

4 Secretary of State for Health and Social Care - UK Government. Coronavirus (COVID-19): notification to organisations to share information. 2022. https://www.gov.uk/government/publications/coronavirus-covid-19-notification-to-

For peer review only - http://bmjopen.bmj.com/site/about/guidelines.xhtml

organisations-to-share-information/coronavirus-covid-19-notice-under-regulation-34-of-the-health-service-control-of-patient-information-regulations-2002

5 Confidentiality Advisory Group. Health Research Authority. https://www.hra.nhs.uk/about-us/committees-and-services/confidentiality-advisory-group/

Data access

Access to the underlying identifiable and potentially re-identifiable pseudonymised electronic health record data is tightly governed by various legislative and regulatory frameworks, and restricted by best practice. The data in OpenSAFELY is drawn from General Practice data across England where EMIS and TPP are the data processors.

EMIS and TPP developers initiate an automated process to create pseudonymised records in the core OpenSAFELY database, which are copies of key structured data tables in the identifiable records. These pseudonymised records are linked onto key external data resources that have also been pseudonymised via SHA-512 one-way hashing of NHS numbers using a shared salt. Bennett Institute for Applied Data Science developers and PIs holding contracts with NHS England have access to the OpenSAFELY pseudonymised data tables as needed to develop the OpenSAFELY tools.

These tools in turn enable researchers with OpenSAFELY data access agreements to write and execute code for data management and data analysis without direct access to the underlying raw pseudonymised patient data, and to review the outputs of this code. All code for the full data management pipeline—from raw data to completed results for this analysis—and for the OpenSAFELY platform as a whole is available for review at github.com/OpenSAFELY.

The data management and analysis code for this paper was led by Dr Mark Green and contributed to by all named authors.

For peer review only

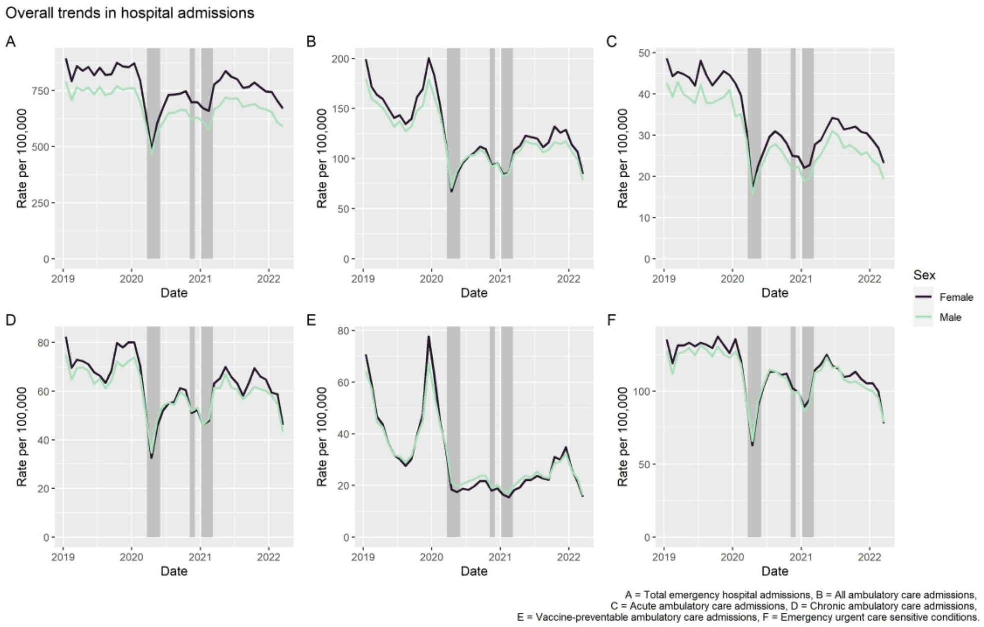

Figure 1

304x191mm (300 x 300 DPI)

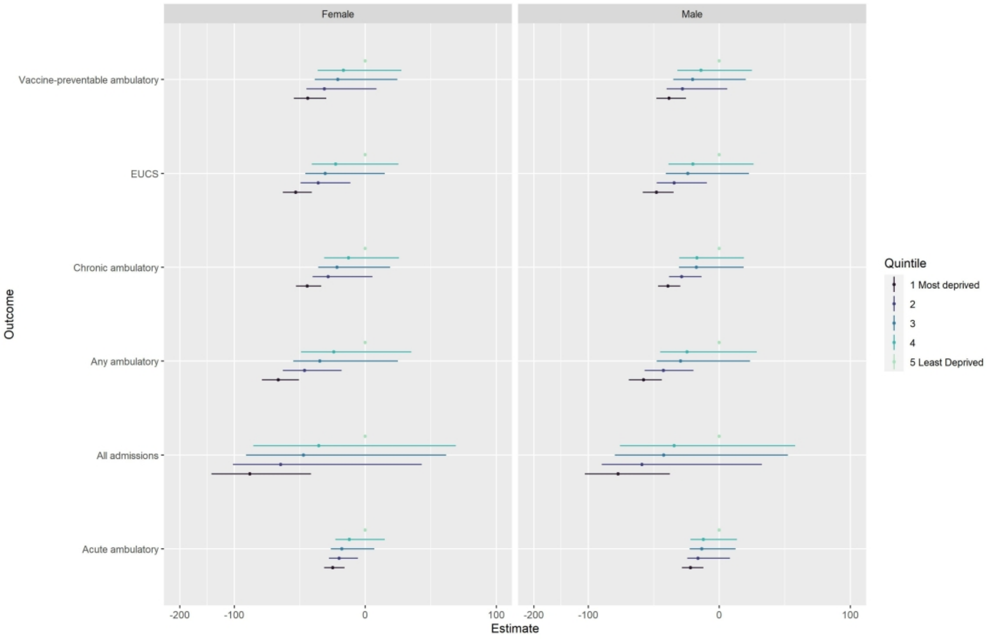

Figure 2

315x202mm (300 x 300 DPI)

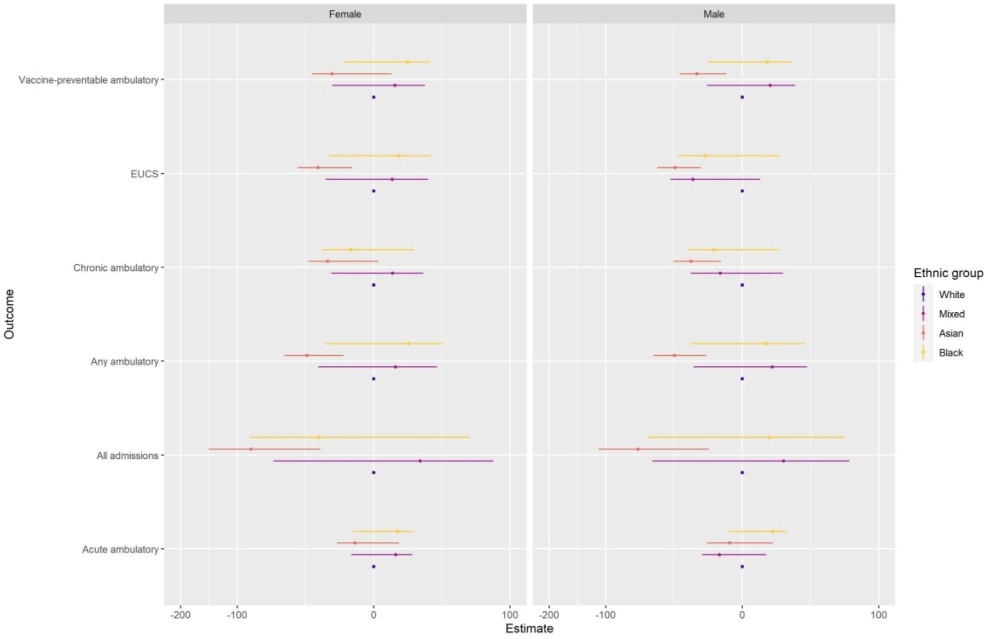

Figure 3

315x202mm (300 x 300 DPI)

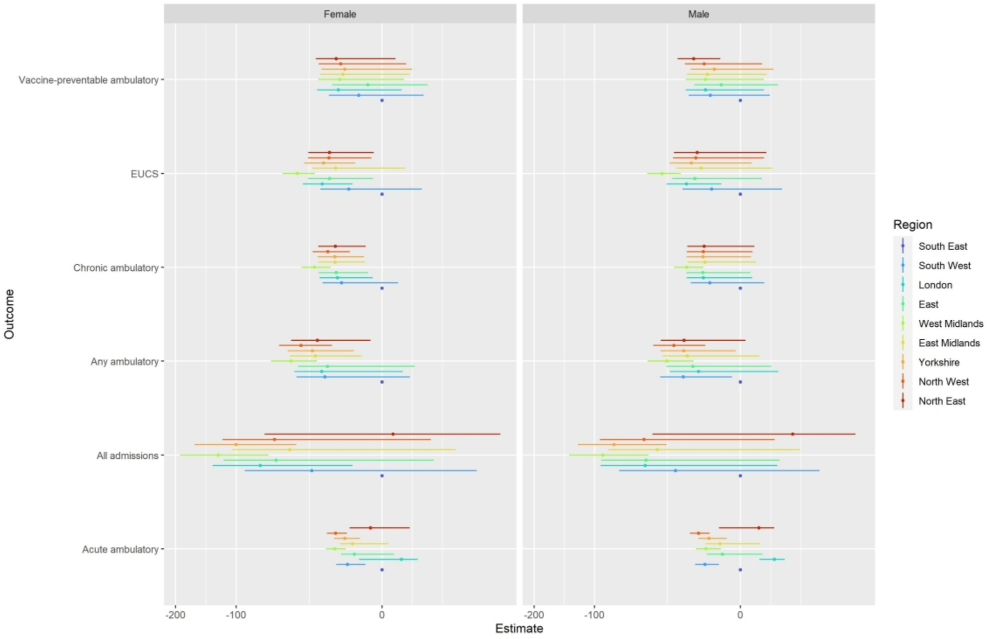

Figure 4

315x202mm (300 x 300 DPI)

Appendix

Table A: Missing data for covariates

Measure	Percent missing (%)
Index of Multiple Deprivation	1.68
Ethnicity	10.21
Government Office Region	0.05

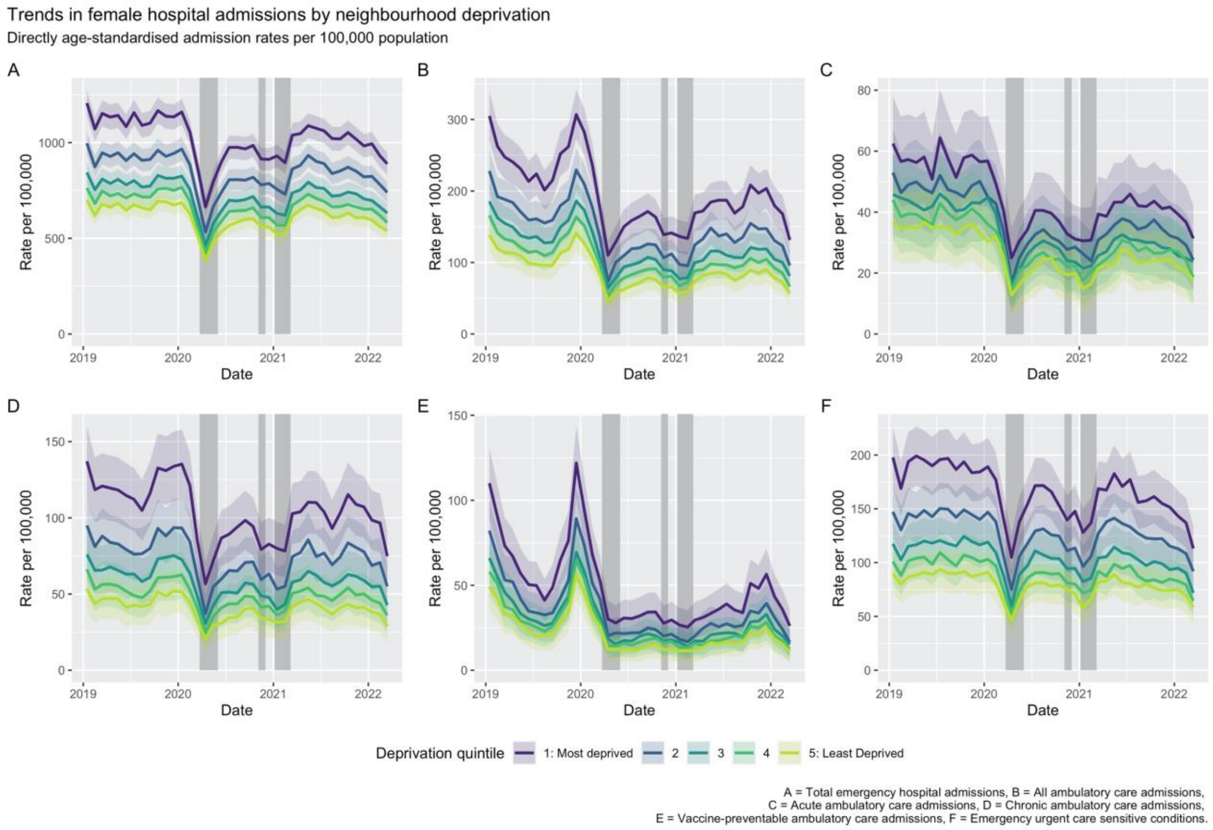

Figure A: Directly age-standardised admission rates for females by socioeconomic deprivation quintile. Note: shaded periods represent national lockdowns.

For peer review only - http://bmjopen.bmj.com/site/about/guidelines.xhtml

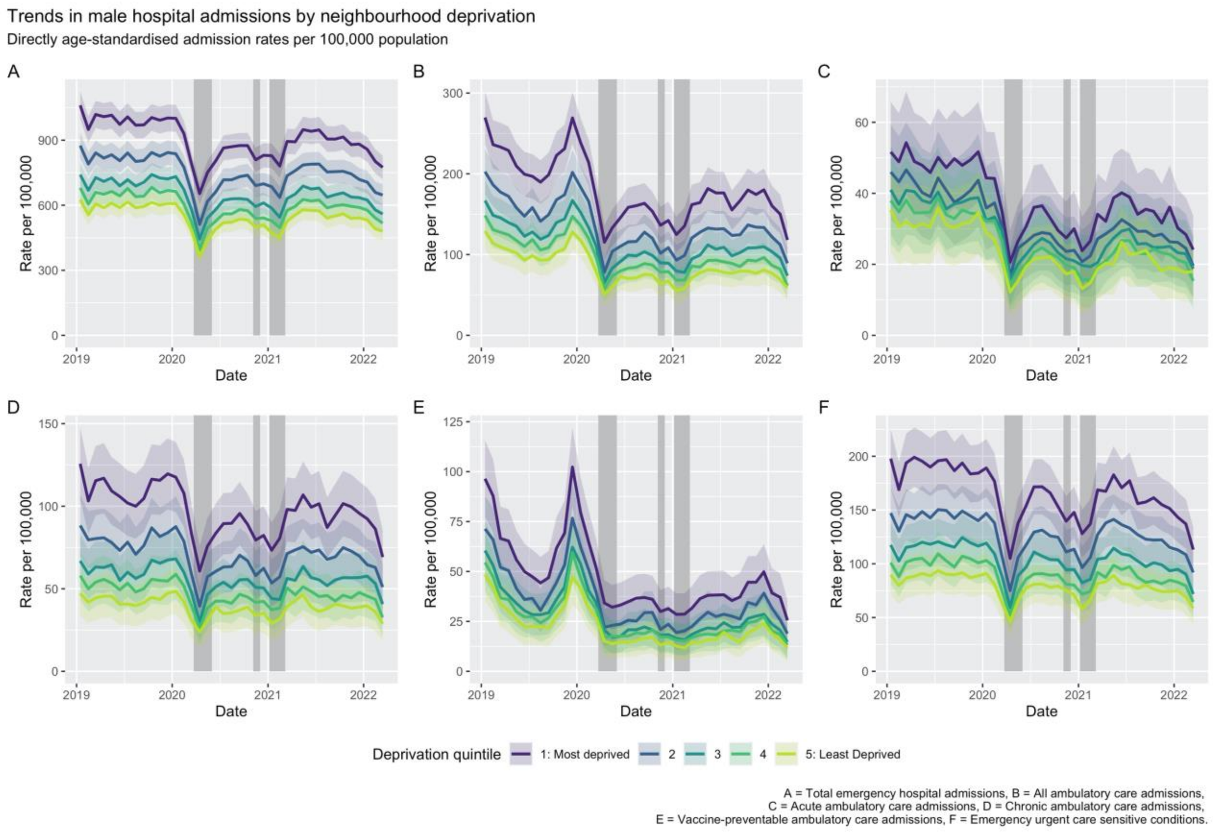

Figure B: Directly age-standardised admission rates for males by socioeconomic deprivation quintile. e: shaded periods represent national lockdowns.

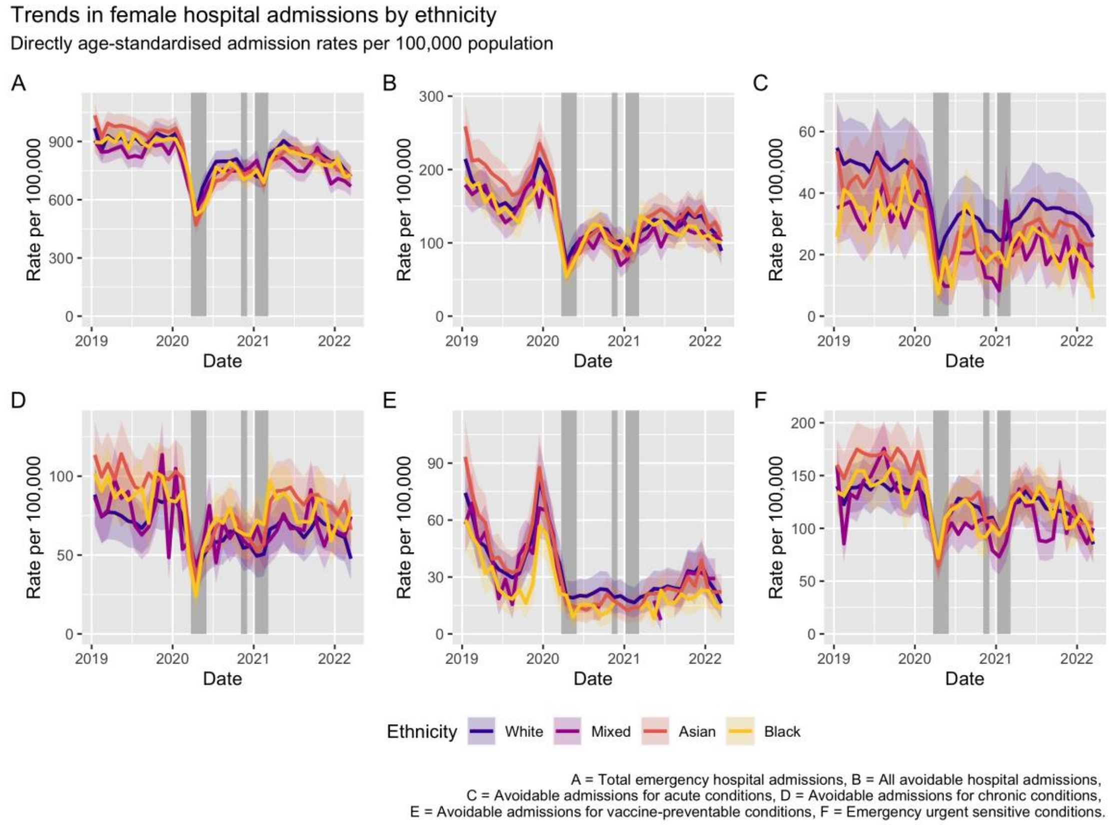

Figure C: Directly age-standardised admission rates for females by ethnicity. e: shaded periods represent national lockdowns.

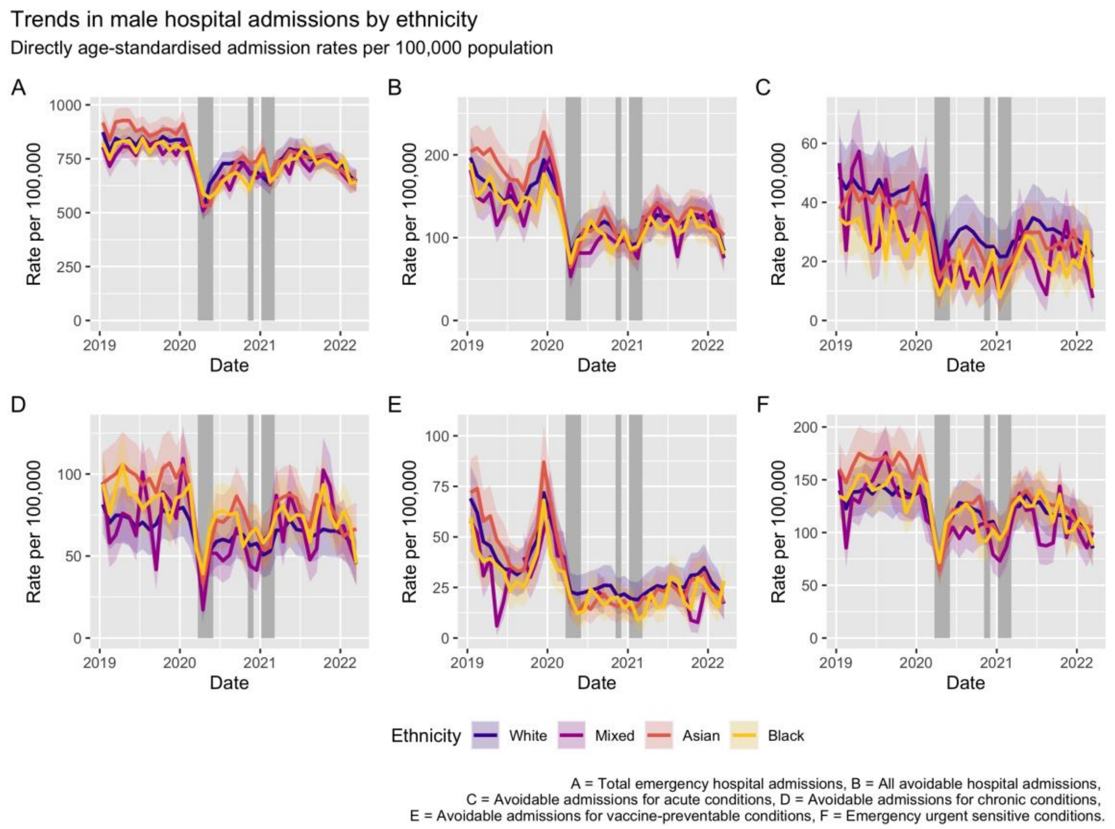

Figure D: Directly age-standardised admission rates for males by ethnicity. e: shaded periods represent national lockdowns.

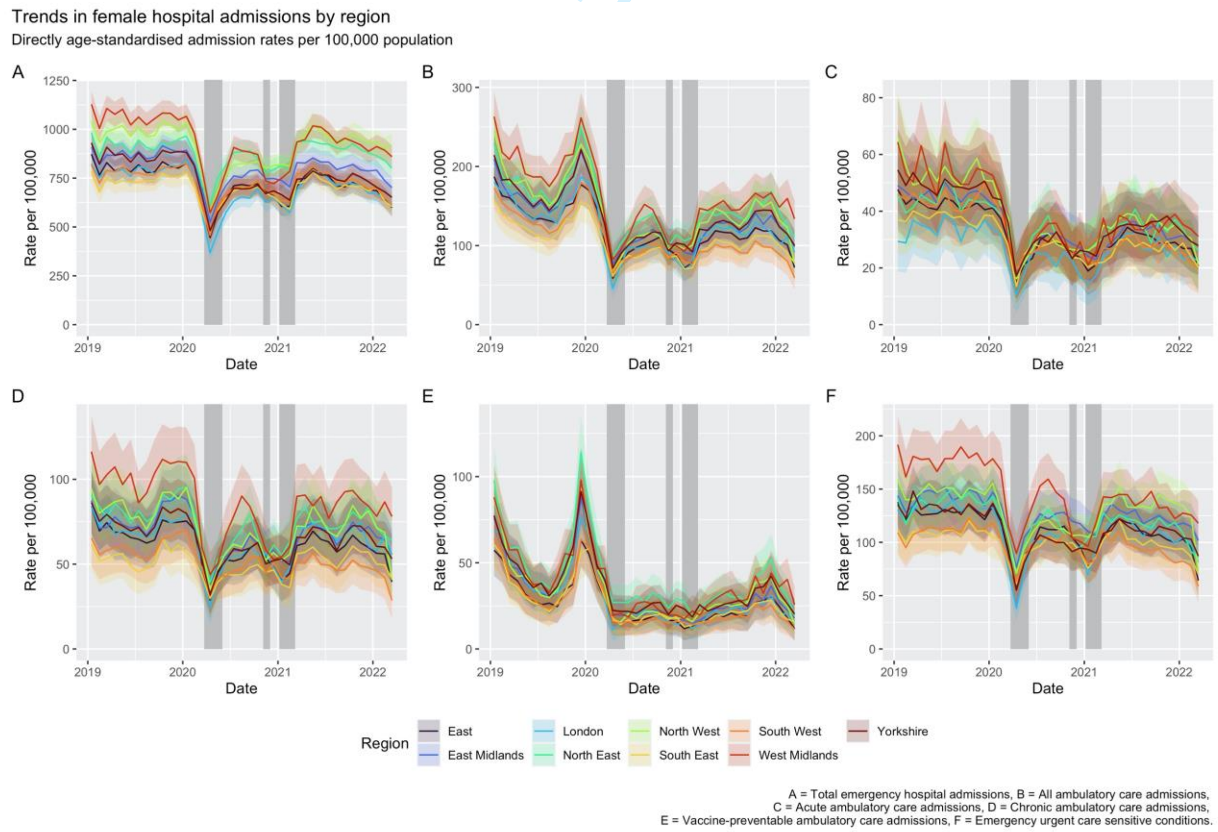

Figure E: Directly age-standardised admission rates for females by region. e: shaded periods represent national lockdowns.

For peer review only - http://bmjopen.bmj.com/site/about/guidelines.xhtml

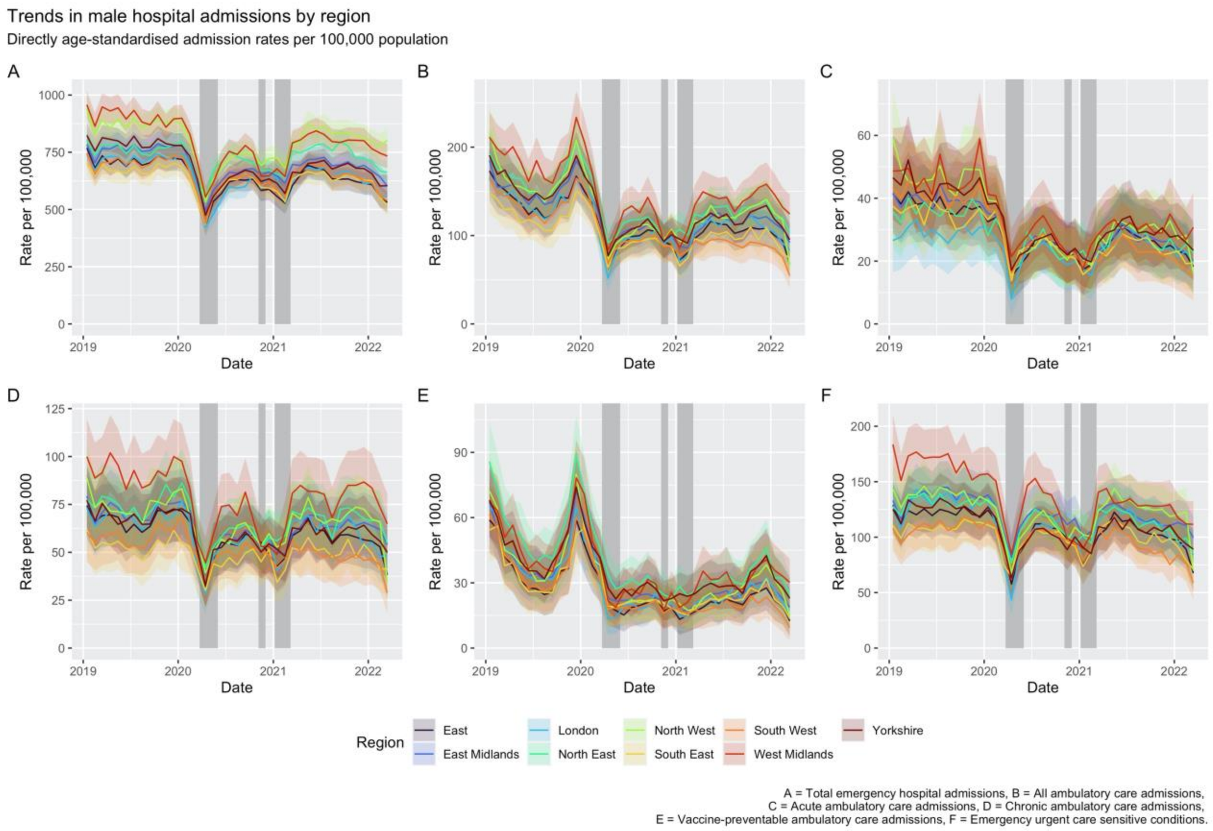

Figure F: Directly age-standardised admission rates for males by region. e: shaded periods represent national lockdowns.

For peer review only - http://bmjopen.bmj.com/site/about/guidelines.xhtml

STROBE 2007 (v4) checklist of items to be included in reports of observational studies in epidemiology*
Checklist for cohort, case-control, and cross-sectional studies (combined)

Section/Topic	Item #	Recommendation	Reported on page #
**Title and abstract**	1	(*a*) Indicate the study's design with a commonly used term in the title or the abstract	1
		(*b*) Provide in the abstract an informative and balanced summary of what was done and what was found	2
**Introduction**			
Background/rationale	2	Explain the scientific background and rationale for the investigation being reported	3
Objectives	3	State specific objectives, including any pre-specified hypotheses	4
**Methods**			
Study design	4	Present key elements of study design early in the paper	4
Setting	5	Describe the setting, locations, and relevant dates, including periods of recruitment, exposure, follow-up, and data collection	4
Participants	6	(*a*) *Cohort study*—Give the eligibility criteria, and the sources and methods of selection of participants. Describe methods of follow-up<br>*Case-control study*—Give the eligibility criteria, and the sources and methods of case ascertainment and control selection. Give the rationale for the choice of cases and controls<br>*Cross-sectional study*—Give the eligibility criteria, and the sources and methods of selection of participants	4
		(*b*) *Cohort study*—For matched studies, give matching criteria and number of exposed and unexposed<br>*Case-control study*—For matched studies, give matching criteria and the number of controls per case	
Variables	7	Clearly define all outcomes, exposures, predictors, potential confounders, and effect modifiers. Give diagnostic criteria, if applicable	4-5
Data sources/ measurement	8*	For each variable of interest, give sources of data and details of methods of assessment (measurement). Describe comparability of assessment methods if there is more than one group	4-5
Bias	9	Describe any efforts to address potential sources of bias	5
Study size	10	Explain how the study size was arrived at	4
Quantitative variables	11	Explain how quantitative variables were handled in the analyses. If applicable, describe which groupings were chosen and why	4-5
Statistical methods	12	(*a*) Describe all statistical methods, including those used to control for confounding	5
		(*b*) Describe any methods used to examine subgroups and interactions	5
		(*c*) Explain how missing data were addressed	5
		(*d*) *Cohort study*—If applicable, explain how loss to follow-up was addressed<br>*Case-control study*—If applicable, explain how matching of cases and controls was addressed	N/A

For peer review only

		Cross-sectional study—If applicable, describe analytical methods taking account of sampling strategy	
		(*e*) Describe any sensitivity analyses	N/A
**Results**			
Participants	13*	(a) Report numbers of individuals at each stage of study—eg numbers potentially eligible, examined for eligibility, confirmed eligible, included in the study, completing follow-up, and analysed	5-6
		(b) Give reasons for non-participation at each stage	N/A
		(c) Consider use of a flow diagram	N/A
Descriptive data	14*	(a) Give characteristics of study participants (eg demographic, clinical, social) and information on exposures and potential confounders	5-6
		(b) Indicate number of participants with missing data for each variable of interest	5
		(c) *Cohort study*—Summarise follow-up time (eg, average and total amount)	N/A
Outcome data	15*	*Cohort study*—Report numbers of outcome events or summary measures over time	N/A
		Case-control study—Report numbers in each exposure category, or summary measures of exposure	N/A
		Cross-sectional study—Report numbers of outcome events or summary measures	5-6
Main results	16	(*a*) Give unadjusted estimates and, if applicable, confounder-adjusted estimates and their precision (eg, 95% confidence interval). Make clear which confounders were adjusted for and why they were included	6-8
		(*b*) Report category boundaries when continuous variables were categorized	N/A
		(*c*) If relevant, consider translating estimates of relative risk into absolute risk for a meaningful time period	N/A
Other analyses	17	Report other analyses done—eg analyses of subgroups and interactions, and sensitivity analyses	6-8
**Discussion**			
Key results	18	Summarise key results with reference to study objectives	8
Limitations	19	Discuss limitations of the study, taking into account sources of potential bias or imprecision. Discuss both direction and magnitude of any potential bias	10
Interpretation	20	Give a cautious overall interpretation of results considering objectives, limitations, multiplicity of analyses, results from similar studies, and other relevant evidence	8-10
Generalisability	21	Discuss the generalisability (external validity) of the study results	11
**Other information**			
Funding	22	Give the source of funding and the role of the funders for the present study and, if applicable, for the original study on which the present article is based	13

*Give information separately for cases and controls in case-control studies and, if applicable, for exposed and unexposed groups in cohort and cross-sectional studies.
Note: An Explanation and Elaboration article discusses each checklist item and gives methodological background and published examples of transparent reporting. The STROBE checklist is best used in conjunction with this article (freely available on the Web sites of PLoS Medicine at http://www.plosmedicine.org/, Annals of Internal Medicine at http://www.annals.org/, and Epidemiology at http://www.epidem.com/). Information on the STROBE Initiative is available at www.strobe-statement.org.

For peer review only

BMJ Open

Trends in inequalities in avoidable hospitalisations across the COVID-19 pandemic: A cohort study of 23.5 million people in England

Journal:	*BMJ Open*
Manuscript ID	bmjopen-2023-077948.R1
Article Type:	Original research
Date Submitted by the Author:	15-Nov-2023

Complete List of Authors:	Green, Mark; University of Liverpool, Geography & Planning<br>McKee, Martin; European Centre on Health of Societ,<br>Massey, Jon; Oxford University, Nuffield Department of Primary Care Health Sciences<br>Mackenna, Brian; NHS England, Medicines and Diagnostics Policy Unit<br>Mehrkar, Amir; Oxford University, Nuffield Department of Primary Care Health Sciences<br>Bacon, Seb; University of Oxford, Nuffield Department of Primary Care Health Sciences<br>Macleod, John; University of Bristol<br>Sheikh, Aziz; University of Edinburgh, Division of Community Health Sciences<br>Shah, Syed Ahmar; The University of Edinburgh Usher Institute of Population Health Sciences and Informatics,<br>Consortium, The OpenSAFELY ; Oxford University<br>National Core Study Collaborative, The Longitudinal Health and Wellbeing; UCL<br>Katikireddi, Srinivasa; University of Glasgow, MRC/CSO Social & Public Health Sciences Unit

**Primary Subject Heading**:	Public health
Secondary Subject Heading:	Epidemiology
Keywords:	Quality in health care < HEALTH SERVICES ADMINISTRATION & MANAGEMENT, Rationing < HEALTH SERVICES ADMINISTRATION & MANAGEMENT, Health policy < HEALTH SERVICES ADMINISTRATION & MANAGEMENT, Public health < INFECTIOUS DISEASES, Population Dynamics, PUBLIC HEALTH

SCHOLARONE™
Manuscripts

I, the Submitting Author has the right to grant and does grant on behalf of all authors of the Work (as defined in the below author licence), an exclusive licence and/or a non-exclusive licence for contributions from authors who are: i) UK Crown employees; ii) where BMJ has agreed a CC-BY licence shall apply, and/or iii) in accordance with the terms applicable for US Federal Government officers or employees acting as part of their official duties; on a worldwide, perpetual, irrevocable, royalty-free basis to BMJ Publishing Group Ltd ("BMJ") its licensees and where the relevant Journal is co-owned by BMJ to the co-owners of the Journal, to publish the Work in this journal and any other BMJ products and to exploit all rights, as set out in our licence.

The Submitting Author accepts and understands that any supply made under these terms is made by BMJ to the Submitting Author unless you are acting as an employee on behalf of your employer or a postgraduate student of an affiliated institution which is paying any applicable article publishing charge ("APC") for Open Access articles. Where the Submitting Author wishes to make the Work available on an Open Access basis (and intends to pay the relevant APC), the terms of reuse of such Open Access shall be governed by a Creative Commons licence – details of these licences and which Creative Commons licence will apply to this Work are set out in our licence referred to above.

Other than as permitted in any relevant BMJ Author's Self Archiving Policies, I confirm this Work has not been accepted for publication elsewhere, is not being considered for publication elsewhere and does not duplicate material already published. I confirm all authors consent to publication of this Work and authorise the granting of this licence.

For peer review only

Trends in inequalities in avoidable hospitalisations across the COVID-19 pandemic: A cohort study of 23.5 million people in England

Mark A. Green[1,2], Martin McKee[3,4], Jon Massey[5,6], Brian MacKenna[5,7], Amir Mehrkar[5,6], Sebastian Bacon[5,6], John Macleod[8,9], Aziz Sheikh[10], Syed Ahmar Shah[10], The OpenSAFELY Consortium, The LH&W NCS Collaborative and Srinivasa Vittal Katikireddi[2].

[1] Geographic Data Science Lab, Department of Geography & Planning, University of Liverpool, Liverpool, UK.
[2] MRC/CSO Social and Public Health Sciences Unit, University of Glasgow, Glasgow, UK.
[3] Department of Health Services Research and Policy, London School of Hygiene and Tropical Medicine, London, UK.
[4] UCL Health Partners, London, UK.
[5] Bennett Institute for Applied Data Science, University of Oxford, Oxford, UK.
[6] Nuffield Department of Primary Care Health Sciences, University of Oxford, Oxford, UK.
[7] NHS England Medicines and Diagnostics Policy Unit, NHS England, London, UK.
[8] Population Health Sciences, University of Bristol, Bristol, UK.
[9] The National Institute for Health and Care Research Applied Research Collaboration West (NIHR ARC West) at University Hospitals Bristol and Weston NHS Foundation Trust, UK
[10] Usher Institute, University of Edinburgh, Edinburgh, UK.

Corresponding author
Mark Green. Address: Roxby Building, University of Liverpool, Liverpool, L69 7ZT. Email: mark.green@liverpool.ac.uk. Tel: +44 151 794 2854.

For peer review only - http://bmjopen.bmj.com/site/about/guidelines.xhtml

ABSTRACT

Objective: To determine whether periods of disruption were associated with increased 'avoidable' hospital admissions and wider social inequalities in England.

Design: Observational repeated cross-sectional study.

Setting: England (January 2019 to March 2022).

Participants: With the approval of NHS England we utilised individual-level electronic health records from OpenSAFELY, which covered ~40% of general practices in England (mean monthly population size 23.5 million people).

Primary and secondary outcome measures: We estimated crude and directly age-standardised rates for potentially preventable unplanned hospital admissions: ambulatory care sensitive conditions and urgent emergency sensitive conditions. We considered how trends in these outcomes varied by three measures of social and spatial inequality: neighbourhood socioeconomic deprivation, ethnicity, and geographical region.

Results: There were large declines in avoidable hospitalisations during the first national lockdown (March-May 2020). Trends increased post-lockdown but never reached 2019 levels. The exception to these trends was for vaccine-preventable ambulatory care sensitive admissions which remained low throughout 2020-2021. While trends were consistent by each measure of inequality, absolute levels of inequalities narrowed across levels of neighbourhood socioeconomic deprivation, Asian ethnicity (compared to White ethnicity), and geographical region (especially in northern regions).

Conclusions: We found no evidence that periods of healthcare disruption from the COVID-19 pandemic resulted in more avoidable hospitalisations. Falling avoidable hospital admissions has coincided with declining inequalities most strongly by level of deprivation, but also for Asian ethnic groups and northern regions of England.

Keywords

COVID-19; avoidable hospitalisations; electronic health records; inequality; health systems; disruption.

Strengths and weaknesses

- Utilises electronic health records for ~23.5M people which are representative of England.
- Examines how trends in avoidable hospitalisations vary across three measures of social and spatial inequality (deprivation, ethnic group and geographic region).
- Analyses are descriptive and unable to explain what factors were driving trends.
- It is uncertain whether avoidable hospitalisations are a good proxy measure for capturing the impacts of healthcare disruption.

For peer review only - http://bmjopen.bmj.com/site/about/guidelines.xhtml

INTRODUCTION

There has been public, political, and media concern that the disruption in healthcare during the pandemic would increase hospital admissions among people not receiving necessary care. Visits to general practice fell markedly (1), although this was compensated for by a online consultations (2). Elective treatments in England in 2020 fell by three million compared to 2019 (3). Cancer screening programmes, non-essential surgeries and diagnostic procedures were postponed or cancelled (1,4). Waiting lists grew, delaying access to care for new treatments (5,6). High levels of staff illness from acute COVID-19 and Long COVID have disrupted care delivered (7). These trends have been observed not only in England, but also many countries (8,9). There has been little empirical evaluation into whether such disruption translated into more hospital admissions. We only know of one study demonstrating that people who self-reported that disruption of access to healthcare had higher odds of admission for a potentially preventable condition in the first two and a half years of the pandemic (10).

Identifying the impacts of healthcare disruption is difficult. The journey from onset of illness to accessing care is complex and obstacles can arise at many points across the health system (11). To simplify this issue, we borrowed the concept 'avoidable hospital admissions' from the health systems literature, considered a proxy measure of health system performance (12). Avoidable hospitalisations are emergency (unplanned) hospital admissions that could potentially have been prevented if individuals had received timely care within the community (and therefore may be susceptible to disruptions to care) (12,13). Reducing avoidable hospital admissions is a priority for the NHS because they are often costly and disrupt elective care (14). Significant disruption to healthcare access, coincing with uncontrolled infection and non-pharmaceutical interventions implemented in response (e.g., people unable to see their GP or postponement to treatments) may have resulted in more avoidable hospitalisations where individuals were not able to receive the care they needed (10).

Research has showed that avoidable hospitalisations in many countries fell in 2020 during the first COVID-19 waves and did not return to pre-pandemic levels thereafter (15–18). Most of this research, however, stops at the end of 2020 and there is less understanding of how trends have continued since. This ignores a critical period in the response and recovery from the pandemic. 2021-2022 saw significant disruption to health systems due to COVID-19 (19). It is also plausible that the impacts of disrupted access to healthcare are not immediate and may only be observable in the medium-term (e.g., delayed diagnosis or treatment leading to disease progression that results in an avoidable hospitalisation a year down the line) (10). Much of this research has been undertaken using small datasets for specific geographic contexts (e.g. single cities or states) which may not be generalisable to the national level. Few studies have examined how trends in avoidable hospitalisations have varied across measures of social inequalities. This is pertinent since the COVID-19 pandemic shined a light on the fractures within English society (20), with people of lower socioeconomic position, minoritised ethnic groups, and those living in parts of northern England disproportionally affected by COVID-19 (21–23). These same communities have not just only been shown to report greater experiences of healthcare disruption (24), but they are also those which had higher rates of avoidable hospitalisations pre-pandemic too

For peer review only - http://bmjopen.bmj.com/site/about/guidelines.xhtml

(12,25–27). We hypothesise that the COVID-19 pandemic may have widened social inequalities in avoidable hospitalisations.

The aim of our study is to determine whether periods of healthcare disruption were associated with increased ‘avoidable’ hospital admissions and wider social inequalities in England.

METHODS

Data

The primary data source used for the analysis was OpenSAFELY-TPP. OpenSAFELY is an open-source secure health data analysis platform providing access to primary care records, linked to secondary care and mortality records, held by the two largest electronic health record providers for NHS England – Egton Medical Information Systems (EMIS) and The Phoenix Partnership (TPP) which cover ~58 million registered patients. In this study, we had access to data from TPP (The Phoenix Partnership) which covers ~40% of general practices in England. The data are broadly representative of England by age, sex, ethnicity, small area socioeconomic deprivation and cause of death (28).

Data were accessed on 20th May 2022. The study period for our analysis was 1st January 2019 to 31st March 2022. While 2019 only provides one year’s worth of pre-pandemic data, overall trends prior to 2019 were relatively flat (29). We discounted all data in April and May 2022 to minimise under-counting of events due to possible delays in reporting of clinical information relating to admissions. Total counts per month for all outcomes were rounded to their nearest 5 to minimise risks of patient identification where low counts were evident (a condition in the standard data agreement). As this only adjusted monthly counts by 1-3 units, the impact was relatively minimal. We redacted counts <10 to meet statistical disclosure requirements. All analytical scripts, processed data and outputs are openly available at https://github.com/opensafely/avoidable_hospitalisations_trends.

Outcomes

Avoidable hospitalisations were defined using five measures commonly used by NHS England (12). Each were defined using ICD-10 codes (codelists openly available at (30)), primary diagnosis (diagnostic position 1) and for emergency (unplanned) admissions only. All ambulatory care sensitive conditions were selected as an overall proxy for avoidable hospitalisations. Ambulatory care sensitive conditions were defined as conditions that can be treated effectively in the community and should not therefore require hospital admission (12,13,29). We further disaggregated all ambulatory care sensitive conditions into three measures representing the type of condition: (i) acute conditions (e.g., cellulitis, dental caries, rickets, gastric ulcer), (ii) chronic conditions (e.g., hypertension, angina, asthma), (iii) vaccine-preventable conditions (e.g., mumps, measles, influenza). We did not include admissions due to COVID-19 as avoidable or vaccine-preventable conditions as we wanted to examine the indirect impacts of the pandemic (as well as ensuring a consistent set of conditions pre-pandemic). We also included emergency urgent care sensitive conditions as an alternative measure of avoidable hospitalisations. These are acute exacerbations of urgent conditions that will result in hospital admission but that the NHS should be able to treat within the community to minimise the need for hospital care (12,13,29). While

For peer review only - http://bmjopen.bmj.com/site/about/guidelines.xhtml

emergency urgent care sensitive conditions overlap with some ambulatory care sensitive conditions, they also include certain conditions not included elsewhere (such as mental and behavioural conditions and falls). Finally, we also included a measure of all emergency hospital admissions to provide context for our measures.

Measures of social inequality

We considered how trends in our outcome variables varied by three measures of social and spatial inequality that have been widely reported to have been associated with unequal COVID-19 outcomes (20,23). Neighbourhood socioeconomic deprivation was measured using the Index of Multiple Deprivation 2019 (IMD) (31). Individuals were matched to Lower Super Output Area by their home residence in each month and we calculated the quintile of deprivation rank. There were no alternative and more recent data available that could measure neighbourhood deprivation during the full period. Ethnicity was recorded in the electronic health record and we consider four groups (White or White British, Black or Black British, Asian or Asian British, and Mixed ethnicity). We did not undertake further disaggregation because of issues with small numbers. We excluded individuals with 'Other' ethnicity since the group is extremely heterogeneous, limiting our scope to draw conclusions (they represented 2.2% of the entire sample). Patients were allocated to seven Government Office Regions, based on home residence, to capture regional differences. Missing data for covariates are presented in Appendix Table A.

Statistical analyses

We estimated summary statistics for each outcome measure using aggregated counts from individual records. Where aggregated statistics were calculated, we used crude rates to measure monthly hospital admission rates (population was defined as total number of people alive on the 1st of each month within the OpenSAFELY-TPP data). Where we stratified measures by socio-demographic indicators, we estimated directly age-standardised rates to adjust for the different age structures of each group that may confound trends. All measures were stratified by sex (female or male). We visualised rates and presented descriptive statistics to investigate trends.

To quantify and measure the extent of how trends in inequalities have changed over time, we used a difference-in-differences regression model. Difference-in-differences is a commonly used method for evaluating natural experiments as it estimates the average change of time of an outcome following an event. This helps us to quantify how trends have changed since the start of the pandemic. Here we fit a separate linear regression model using the monthly observations for each socio-demographic measure independently as the dataset (i.e., three separate models). The directly age-standardised admission rate of each month was used as our outcome variable, with a binary predictor variable for if the month was pre- or post-pandemic (defined here as March 2020 onwards), a categorical variable for the specific socio-demographic measure of inequality (deprivation quintile, ethnic group or geographic region), and an interaction effect between the binary and categorical variables. Models were stratified by sex. We focus on the interaction effect here since it tells us how the trend changed overall, indicating if inequalities between socio-demographic grows narrowed or widened. We excluded all missing data from analyses.

Patient and Public Involvement

For peer review only - http://bmjopen.bmj.com/site/about/guidelines.xhtml

There was no public engagement in the design or completion of this project.

RESULTS

Overall population-level trends

Table 1 presents key summary statistics for our data. During the study period, there were 6 645 550 emergency hospital admissions, with a monthly average of 170 399. There were 1 129 770 ambulatory care sensitive hospital admissions (17% of all emergency admissions), chronic ambulatory care sensitive hospital admissions being most common. There 1 031 205 emergency urgent care sensitive hospital admissions (16% of all emergency admissions).

Table 1: Frequency counts for outcome measures (1st January 2019 to 31st March 2022).

Measure	Total over study period	Mean per month
Emergency admissions	6,645,550	170,399
All ambulatory care sensitive admissions	1,129,770	28,968
Acute ambulatory care sensitive admissions	292,630	7,503
Chronic ambulatory care sensitive admissions	563,740	14,455
Vaccine-preventable ambulatory care sensitive admissions	282,630	7,247
Emergency urgent care sensitive admissions	1,031,205	26,441
Population	918,302,035	23,546,206

Figure 1 presents crude admission rates by sex over the study period for each of our outcome measures. Trends in emergency hospital admissions were stable and consistent throughout 2019 (Figure 1A). In 2020, there were sudden large falls in emergency admissions coinciding with the first national lockdown in England (e.g., in April 2020 rates were 44% lower (females) and 39% lower (males) than compared to rates in January 2020). Emergency admission rates then increased quickly in the period following the national lockdown, although increases did not reach the same levels as in 2019. Rates fell once more at the end of 2020 (again coinciding with national lockdowns), subsequently recovering quickly and remaining at a level higher in 2021 than in 2020, but still less than in 2019.

Patterns of avoidable hospitalisations largely follow similar trends to emergency hospital admissions. There were large falls in all, acute, and chronic ambulatory care sensitive hospital admissions and emergency urgent care sensitive admissions between March and May 2020. These falls were then followed by large rises, albeit not to 2019 levels, before subsequent falls during the second and third national lockdowns (with large rises following). Vaccine-preventable ambulatory care sensitive admissions were the only measure that did not follow this trend. There were distinct peaks in winter 2019 but, after December, rates fell sharply and then continued at a low level throughout all of 2020. Rates then began to rise throughout 2021 (although only to the lowest points of 2019), peaking in December 2021, before falling thereafter.

For peer review only

For peer review only - http://bmjopen.bmj.com/site/about/guidelines.xhtml

[Figure 1 here]

Figure 1: Crude hospital admission rates (per 100,000 population) by sex for measures of avoidable hospitalisations. Note: shaded periods represent national lockdowns.

Trends by deprivation

We next examined whether trends in our measures varied by deprivation. Appendix Figures A and B present trends for females and males respectively. There were minimal differences by sex so we described the findings together. A distinct social gradient was evident for all outcomes, with the highest rates in the most deprived quintile and lowest in the least deprived quintile. Trends over the study period largely followed those reported at the population level and were consistent across deprivation quintiles.

Figure 2 presents the interaction effects from our difference-in-differences models. The associations are negative which suggest that inequalities by deprivation narrowed over the study period. Only for the most deprived quintile did the 95% confidence intervals not cross a value of 0 for all outcomes. The extent of this narrowing of inequalities was not consistent throughout the study period. The range between most and least deprived quintiles was smallest during the first national lockdown. For example, the range for females was 65 per 100,000 in April 2020 compared to 156 per 100,000 in January 2020. The range of values then increased following the lockdown but always remained smaller than what was observed in 2019. This would support the notion that absolute levels of social inequalities by deprivation have narrowed. However, relative differences remained similar (e.g., 2.4 times higher in April 2020 compared to 2.3 times higher in January 2020 for females) suggesting that inequalities were still important determinants.

[Figure 2]

Figure 2: Estimated interaction effects (with 95% confidence intervals) between start of the pandemic and deprivation quintile from a series of difference-in-differences regression models for six outcome variables. Note: EUCS = Emergency Urgent Care Sensitive admissions. Ambulatory = ambulatory care sensitive admissions. Least deprived quintile was selected as the reference group.

Trends by ethnicity

Appendix Figures C and D present trends by ethnic group and sex. The general trends for each outcome match those described in earlier sections, with the falls in hospital admissions during key COVID-19 waves consistent across all ethnic groups. While there is some ordering of rates by ethnic group, the ordering itself is not consistent and confidence intervals often overlap, suggesting that any ordering is not meaningful. There were no clear observable differences between groups during the main waves/lockdowns.

When estimating how trends changed overall (Figure 3), we did not find evidence of significantly different trends for mixed and black ethnic groups in comparison to white ethnicity. For Asian ethnicity, we find evidence of negative associations which suggests that trends in avoidable hospitalisations declined at a greater rate than compared to the white ethnic group over the period.

[Figure 3 here]

For peer review only - http://bmjopen.bmj.com/site/about/guidelines.xhtml

Figure 3: Estimated interaction effects (with 95% confidence intervals) between start of the pandemic and ethnic group from a series of difference-in-differences regression models for six outcome variables. Note: EUCS = Emergency Urgent Care Sensitive. Ambulatory = ambulatory care sensitive conditions. White ethnicity was selected as the reference group.

Trends by region

Appendix Figures E and F present trends by geographical region for England. Trends largely follow those described previously, with falls in admissions across all outcomes during national lockdowns consistent across all regions. There were noticeable inequalities between regions. Rates in the West Midlands were consistently highest for most outcomes (other than vaccine-preventable care sensitive admissions), followed by North West and North East. Admission rates in the South East and South West were consistently lowest across all periods and for all outcomes. London is the exception, lying in the middle for most outcomes. However, during the first national lockdown (and to a lesser extent at the end of 2020), falling rates take London to the lowest values.

When estimating the overall change in trends, we found inconsistent patterns (Figure 4). Yorkshire and West Midlands had the greatest number of estimates that were statistically significant. Northern regions also saw negative associations in ambulatory care sensitive conditions for females, but not males (in comparison to the South East).

[Figure 4 here]

Figure 4: Estimated interaction effects (with 95% confidence intervals) between start of the pandemic and geographic office region from a series of difference-in-differences regression models for six outcome variables. Note: EUCS = Emergency Urgent Care Sensitive admissions. Ambulatory = ambulatory care sensitive admissions. South East region was selected as the reference group.

DISCUSSION

Key results

Using electronic health records for an average of 23.M people per month, we demonstrate that the COVID-19 pandemic led to a step-change in 'avoidable' hospital admissions. All measures of avoidable hospitalisations we analysed declined during the first national lockdown. While rates increased subsequently (other than in periods of national lockdowns), avoidable hospitalisations have remained lower than 2019 levels. Vaccine-preventable ambulatory care sensitive conditions have remained low for 2020 and 2021, and did not follow trends for other measures of avoidable hospitalisations. Avoidable hospitalisations were highest in the most deprived areas, Yorkshire and West Midlands, and inconsistent across ethnic groups. We find evidence of narrowing absolute levels of inequalities by deprivation, ethnicity, and geographical region. The largest falls in inequalities occurred during the first national lockdown and have remained lower thereafter.

Interpretation

For peer review only - http://bmjopen.bmj.com/site/about/guidelines.xhtml

Our study is one of the largest to investigate how avoidable hospitalisations changed before and during the COVID-19 pandemic. We find large falls in avoidable hospitalisations during periods of national lockdowns. During periods when society opened up, rates increased but never to 2019 levels suggesting a gradual recovery in population-level trends in avoidable hospitalisations. These findings follow similar trends observed in other countries despite their differences in health systems, socio-cultural contexts and experiences of COVID-19 (15–18). We extend on this work through analysing a longer time period and through detailed disaggregated by markers of socioeconomic inequality. Our results also follow observations for other metrics including falls in non-COVID-19 mortality (32,33).

While one interpretation might therefore be that the impacts of healthcare disruption were minimal, we should not forget that healthcare disruption is part of the explanation for the trends we observe. During the first national lockdown, the NHS cancelled or postponed or many patients planning to be admitted to free capacity for patients with COVID-19 (3,4,6). Individuals may also have avoided going to hospital if at all possible for fear of being exposed to SARS-CoV-2 (34). Such disruption may explain why we saw the largest decreases in rates during the first lockdown; the NHS was trying to avoid admitting people to hospital unless events were life threatening or required urgent care. More people dying at home rather than at hospital may have translated into fewer hospital admissions (35), although one study found that people who died at home were more likely to have had an emergency admission during the pandemic (36). Deaths at home also consistently increased across all ethnic groups and levels of deprivation, suggesting that they could not explain the narrowing of inequalities (36). The extent of declines in trends is likely to reflect significant changes in patient behaviour (e.g., fear of being exposed to SARS-CoV-2 in healthcare settings, not wanting to burden services in a crisis) and access to health systems (e.g., physicians deciding not to refer patients to hospital, changes in how admissions are made, prioritisation of community care). Unpicking the exact drivers is complex, especially in how they affected the different dimensions of inequalities, given the numerous interacting factors that may have produced this drop in avoidable hospitalisations and represents an important research gap.

Vaccine-preventable ambulatory care sensitive hospital admissions had markedly different trends to the other types of avoidable hospitalisations. Low levels of vaccine-preventable ambulatory care hospital admissions in 2020-2021 reflects the low level of influenza (37). Influenza normally accounts for a large proportion of these hospital admissions but, as with all respiratory infections, of its transmission is reduced by the non-pharmaceutical interventions adopted for SARS-CoV-2 (38,39). Government advice on shielding and changes in behaviours during the pandemic may also explain these trends. Reductions in other viral and respiratory conditions, such as asthma exacerbations in 2020, also follow the same observed trends (33). However, now that all COVID-19 related non-pharmaceutical interventions have been relaxed, there is a need to prepare for co-occurring waves of SARS-CoV-2 and influenza (40).

The impacts of the COVID-19 pandemic have in many respects been highly unequal (20). While our results provide evidence on the unequal distribution of avoidable hospitalisations across social and spatial characteristics, the changing trends in inequalities are not always so obvious, consistent and often nuanced in their interpretation. Contrary to our hypothesis,

For peer review only - http://bmjopen.bmj.com/site/about/guidelines.xhtml

we find evidence that absolute levels of inequalities in avoidable hospitalisations across deprivation, ethnicity and region narrowed. Counterintuitively for deprivation, the narrowing of inequalities occurred during the first national lockdown, suggesting that the period of greatest disruption in the access to healthcare did not disproportionally impact people in these areas. We are not aware of any specific government interventions that would explain these trends. An evaluation of local and national policies that might have narrowed inequalities would represent important future research. We cannot discount entirely the possibility that this is a consequence of fewer hospital admissions during these periods. It may be that individuals who would have normally been captured in avoidable hospitalisations had experienced COVID-19, been hospitalised and died as a result instead (especially given the disproportionate distribution of unplanned hospitalisation and COVID-19 outcomes by our measures of inequality). However, we did not find evidence that inequalities returned back to 2019 levels during periods of low disruption or outside of COVID-19 waves which would suggest the driver is different.

Although inequalities narrowed, there were still differences between regions, ethnic groups and levels of deprivation reminding us that inequalities remain important. More deprived areas and northern regions had consistently higher avoidable hospitalisations throughout the whole study period. There was a lack of literature on ethnic inequalities in avoidable hospitalisations pre-pandemic (12), and our work contributes to showing how intricate they can be when investigating trends. Trends between Black and Mixed ethnic groups to White ethnicity were not different, which is different to experiences in the USA (16). Our findings reiterate the need for policy makers to focus on tackling the root structural causes of health. Health is socially determined (20). We need to better understand how and why the social determinants of health influence the risk of avoidable hospitalisations to identify areas we can intervene. These need to include both social (e.g., people in more deprived areas working multiple jobs which don't have time to see their GP and end up using more flexible emergency care instead) and spatial factors (e.g., are there cultural or infrastructure reasons that explain why northern regions perform poorly). While we do not find clear and consistent inequalities for ethnicity, this should not detract from the structural injustices that minoritised ethnic groups face which has implications for their health (21).

Limitations
Our analyses are descriptive and are unable to identify the reasons behind what the trends we observe. We did not have access to data pre-2019 that could help to disentangle our findings from longer-term trends. The findings may be subject to ecological fallacy as we had no measure of individual experience of healthcare disruption. However, the magnitude of changes are clear enough to suggest that they are real at least at the population level. While our electronic health records provide large representative data on patients (28), they lack the detail about the contexts of individual's lives that can be found in other data types (e.g., longitudinal surveys) (10).

By using a proxy measure of health system performance, we are also unable to ascertain the extent that our findings do reflect actual disruption to healthcare. Its impacts will be complex, occurring over the short- and long-term. It may be that our study is too early to detect the full impact of this disruption and it will be necessary to continually monitor trends in our outcomes. Additionally, there is some debate in the literature over how valid

For peer review only - http://bmjopen.bmj.com/site/about/guidelines.xhtml

avoidable hospitalisations are as a proxy for health system performance (12). Although our measures are used by NHS England for measuring health system performance (12), other COVID-19 related outcomes have witnessed widening social inequalities which are different to our findings (1,20,21). Regional and hospital differences in reporting patterns may also exist. Validating our chosen outcomes is necessary for understanding the importance of our findings.

We use coarse groupings for each of our measures of inequality. For example, we group individuals by broad ethnic groups that hide the diversity within each group (e.g., Asian constitutes communities from very different backgrounds and heritages). This was partly to minimise statistical disclosure issues, due to the lower number of events per month in some groups. Redacted counts (<10) only affected the ethnicity results for vaccine-preventable ambulatory care sensitive conditions where 69 monthly values (17.6%) were redacted. 61 of these data points were for the 'mixed' ethnic group between April 2020 and August 2021. No other outcomes and exposures were affected. Future research should explore further the scale and nature of the extent of inequalities, involving describing intersectional characteristics across age, sex, ethnicity, deprivation and region. Ideally, this would be hypothesis driven to avoid the risk of spurious associations. Similarly, moving beyond describing geographical inequalities by region to identify how trends vary across smaller places can help to present more precise patterns and help to identify potential drivers.

CONCLUSIONS

The COVID-19 pandemic has affected avoidable hospitalisations in unexpected and potentially surprising ways. Worries that the pandemic would see rising hospital admissions due to disruptions in accessing care do not appear to have materialised, at least so far. While social and spatial inequalities narrowed during the pandemic, recent widening regional inequalities present cause for concern and present the case for renewed focus among narratives of 'building back better' and 'levelling up'.

REFERENCES

1. Mansfield KE, Mathur R, Tazare J, Henderson AD, Mulick AR, Carreira H, et al. Indirect acute effects of the COVID-19 pandemic on physical and mental health in the UK: a population-based study. Lancet Digit Heal. 2021;3(4):e217–30.
2. Green MA, McKee M, Katikireddi SV. Remote general practitioner consultations during COVID-19. Lancet Digit Heal. 2022;4(1):e7.
3. Warner M, Burn S, Stoye G, Aylin PP, Bottle A, Propper C. Socioeconomic deprivation and ethnicity inequalities in disruption to NHS hospital admissions during the COVID-19 pandemic: a national observational study. BMJ Qual Saf. 2022;31(8):590–8.
4. Maringe C, Spicer J, Morris M, Purushotham A, Nolte E, Sullivan R, et al. The impact of the COVID-19 pandemic on cancer deaths due to delays in diagnosis in England, UK: a national, population-based, modelling study. Lancet Oncol. 2020;21(8):1023–34.
5. BMA. NHS backlog data analysis 2022. 2022. Available from: https://www.bma.org.uk/advice-and-support/nhs-delivery-and-workforce/pressures/nhs-backlog-data-analysis
6. Gardner T, Fraser C. Longer waits, missing patients and catching up. 2021. Available from: https://www.health.org.uk/news-and-comment/charts-and-infographics/how-is-elective-care-coping-with-the-continuing-impact-of-covid-19

For peer review only - http://bmjopen.bmj.com/site/about/guidelines.xhtml

7. McKee M, Hiam L. Britain's not working. BMJ. 2022;378:o1883.
8. COVIDSurg Collaborative. Effect of COVID-19 pandemic lockdowns on planned cancer surgery for 15 tumour types in 61 countries: an international, prospective, cohort study. Lancet Oncol. 2021;22(11):1507–17.
9. Webb E, Hernández-Quevedo C, Williams G, Scarpetti G, Reed S, Panteli D. Providing health services effectively during the first wave of COVID-19: A cross-country comparison on planning services, managing cases, and maintaining essential services. Health Policy. 2022;126(5):382–90.
10. Green MA, McKee M, Hamilton O, Shaw RJ, Macleod J, Boyd A, et al. Associations between self-reported healthcare disruption due to covid-19 and avoidable hospital admission: evidence from seven linked longitudinal studies for England. BMJ. 2023;382:e075133.
11. Seguin ML, Rangnekar A, Renedo A, Palafox B, McKee M, Balabanova D. Systematic review of frameworks used to conceptualise health pathways of individuals diagnosed with cardiovascular diseases. BMJ Glob Heal. 2020;5(9):e002464.
12. O'Cathain A, Knowles E, Turner J, Maheswaran R, Goodacre S, Hirst E, et al. Explaining variation in emergency admissions: a mixed methods study of emergency and urgent care systems. Heal Serv Deliv Res. 2014;2:48.
13. Coleman P, Nicholl J. Consensus methods to identify a set of potential performance indicators for systems of emergency and urgent care. J Health Serv Res Policy. 2010;15 Suppl 2:12–8.
14. NHS England. The NHS Long Term Plan. 2019. Available from: https://www.longtermplan.nhs.uk/publication/nhs-long-term-plan/
15. Becker N V, Karmakar M, Tipirneni R, Ayanian JZ. Trends in Hospitalizations for Ambulatory Care-Sensitive Conditions During the COVID-19 Pandemic. JAMA Netw open. 2022;5(3):e222933.
16. Leuchter RK, Villaflores CWA, Norris KC, Sorensen A, Vangala S, Sarkisian CA. Racial Disparities in Potentially Avoidable Hospitalizations During the COVID-19 Pandemic. Am J Prev Med. 2021;61(2):235–9.
17. Rennert-May E, Leal J, Thanh NX, Lang E, Dowling S, Manns B, et al. The impact of COVID-19 on hospital admissions and emergency department visits: A population-based study. PLoS One. 2021;16(6):e0252441.
18. Abe K, Kawachi I, Iba A, Miyawaki A. In-Hospital Deaths From Ambulatory Care–Sensitive Conditions Before and During the COVID-19 Pandemic in Japan. JAMA Netw Open. 2023;6(6):e2319583–e2319583.
19. Green MA, Hungerford DJ, Hughes DM, Garcia-Fiñana M, Turtle L, Cheyne C, et al. Changing patterns of SARS-CoV-2 infection through Delta and Omicron waves by vaccination status, previous infection and neighbourhood deprivation: a cohort analysis of 2.7 M people. BMC Infect Dis. 2022;22(1):889.
20. Bambra C, Riordan R, Ford J, Matthews F. The COVID-19 pandemic and health inequalities. J Epidemiol Community Heal. 2020;74(11):964–8.
21. Mathur R, Rentsch CT, Morton CE, Hulme WJ, Schultze A, MacKenna B, et al. Ethnic differences in SARS-CoV-2 infection and COVID-19-related hospitalisation, intensive care unit admission, and death in 17 million adults in England: an observational cohort study using the OpenSAFELY platform. Lancet. 2021;397(10286):1711–24.
22. Griffith GJ, Owen G, Manley D, Howe LD, Davey Smith G. Continuing inequalities in COVID-19 mortality in England and Wales, and the changing importance of regional,

For peer review only

For peer review only - http://bmjopen.bmj.com/site/about/guidelines.xhtml

over local, deprivation. Health Place. 2022;76:102848.
23. UK Government. Final report on progress to address COVID-19 health inequalities. 2021. Available from: https://www.gov.uk/government/publications/final-report-on-progress-to-address-covid-19-health-inequalities
24. Maddock J, Parsons S, Di Gessa G, Green MJ, Thompson EJ, Stevenson AJ, et al. Inequalities in healthcare disruptions during the Covid-19 pandemic: Evidence from 12 UK population-based longitudinal studies. BMJ Open. 2021;12:e064981.
25. Jivraj S, Murray ET, Norman P, Nicholas O. The impact of life course exposures to neighbourhood deprivation on health and well-being: a review of the long-term neighbourhood effects literature. Eur J Public Health. 2020;30(5):922–8.
26. Katikireddi SV, Cezard G, Bhopal RS, Williams L, Douglas A, Millard A, et al. Assessment of health care, hospital admissions, and mortality by ethnicity: population-based cohort study of health-system performance in Scotland. Lancet Public Heal. 2018;3(5):e226–36.
27. Westbury LD, Syddall HE, Simmonds SJ, Cooper C, Sayer AA. Identification of risk factors for hospital admission using multiple-failure survival models: a toolkit for researchers. BMC Med Res Methodol. 2016;16:46.
28. Andrews C, Schultze A, Curtis H, Hulme W, Tazare J, Evans S, et al. OpenSAFELY: Representativeness of electronic health record platform OpenSAFELY-TPP data compared to the population of England. Wellcome open Res. 2022;7:191.
29. Nuffield Trust. Potentially preventable emergency admissions. 2022. Available from: https://www.nuffieldtrust.org.uk/resource/potentially-preventable-emergency-hospital-admissions
30. Green MA. Open Codelists. 2022. Available from: https://www.opencodelists.org/users/mgreen/
31. Ministry of Housing Communities and Local Government. The English Indices of Deprivation 2019. 2019. Available from: https://www.gov.uk/government/statistics/english-indices-of-deprivation-2019
32. Tallack C, Krelle H. What has happened to non-COVID mortality during the pandemic? 2021. Available from: https://www.health.org.uk/publications/long-reads/what-has-happened-to-non-covid-mortality-during-the-pandemic
33. Shah SA, Brophy S, Kennedy J, Fisher L, Walker A, Mackenna B, et al. Impact of first UK COVID-19 lockdown on hospital admissions: Interrupted time series study of 32 million people. eClinicalMedicine. 2022;49.
34. Watt T, Sullivan R, Aggarwal A. Primary care and cancer: an analysis of the impact and inequalities of the COVID-19 pandemic on patient pathways. BMJ Open. 2022;12(3):e059374.
35. ONS. Deaths at home increased by a third in 2020, while deaths in hospitals fell except for COVID-19. 2021. Available from: https://www.ons.gov.uk/peoplepopulationandcommunity/birthsdeathsandmarriages/deaths/articles/deathsathomeincreasedbyathirdin2020whiledeathsinhospitalsfellexceptforcovid19/2021-05-07
36. Keeble E, Scobie S, Georghiou T, Davies M. Deaths at home during the Covid-19 pandemic and implications for patients and services. 2023. Available from: https://www.nuffieldtrust.org.uk/research/deaths-at-home-during-the-covid-19-pandemic-and-implications-for-patients-and-services
37. UK Health Security Agency. Surveillance of influenza and other seasonal respiratory

For peer review only - http://bmjopen.bmj.com/site/about/guidelines.xhtml

viruses in winter 2021 to 2022. 2022. Available from: https://www.gov.uk/government/statistics/annual-flu-reports/surveillance-of-influenza-and-other-seasonal-respiratory-viruses-in-winter-2021-to-2022
38. Huang QS, Wood T, Jelley L, Jennings T, Jefferies S, Daniells K, et al. Impact of the COVID-19 nonpharmaceutical interventions on influenza and other respiratory viral infections in New Zealand. Nat Commun. 2021;12:1001.
39. Davis WW, Mott JA, Olsen SJ. The role of non-pharmaceutical interventions on influenza circulation during the COVID-19 pandemic in nine tropical Asian countries. Influenza Other Respi Viruses. 2022;16(3):568–76.
40. Nazareth J, Pan D, Martin CA, Barr I, Sullivan SG, Stephenson I, et al. Is the UK prepared for seasonal influenza in 2022-23 and beyond? Lancet Infect Dis. 2022; 22(9):1280-1281.

Competing interests

None declared.

Acknowledgements

We are very grateful for all the support received from the TPP Technical Operations team throughout this work, and for generous assistance from the information governance and database teams at NHS England and the NHS England Transformation Directorate. Information about data sharing, data access and information governance can be found at the end of this document.

Funding

This work was funded as part of the National Core Studies Longitudinal Health & Wellbeing programme (MC_PC_20030, MC_PC_20059). MAG acknowledges funding for this research from UK Medical Research Council (MR/W021242/1). SVK acknowledges funding from a NRS Senior Clinical Fellowship (SCAF/15/02), the Medical Research Council (MC_UU_00022/2) and the Scottish Government Chief Scientist Office (SPHSU17). JM is partly funded by the National Institute for Health and Care Research Applied Research Collaboration West (NIHR ARC West). The full statement, listing the names of all relevant NCS Consortium staff, can be found here: https://www.ucl.ac.uk/covid-19-longitudinal-health-wellbeing/convalescence-study-collaborative.

Ethics

Ethical approval for the project was granted by the University of Liverpool's Research Ethics Board (reference 10634).

Author contributions

MAG, MM and SVS came up with the idea for the study. MAG, MM and SVS designed the study, with input from AS, JM and SAS. JM, BM, AM and SB were involved in data access and collection. MAG and JM manipulated data into an analysis ready format. MAG led on data analyses, supported by SVS. MAG, MM and SVS wrote the initial draft of the paper, with all other authors revising the paper.

Data sharing statement

For peer review only - http://bmjopen.bmj.com/site/about/guidelines.xhtml

All data were linked, stored and analysed securely within the OpenSAFELY platform: https://opensafely.org/. Data include pseudonymised data such as coded diagnoses, medications and physiological parameters. No free text data are included. All code is shared openly for review and re-use under MIT open license on https://github.com/opensafely/avoidable_hospitalisations_trends. Detailed pseudonymised patient data is potentially re-identifiable and therefore not shared. Primary care records managed by the GP software provider, TPP, were linked to Hospital Episode Statistics through OpenSAFELY.

Information governance

NHS England is the data controller for OpenSAFELY-TPP and OpenSAFELY-EMIS; EMIS and TPP are the data processors; all study authors using OpenSAFELY have the approval of NHS England. This implementation of OpenSAFELY is hosted within the EMIS and TPP environments which are accredited to the ISO 27001 information security standard and are NHS IG Toolkit compliant.[1]

Patient data has been pseudonymised for analysis and linkage using industry standard cryptographic hashing techniques; all pseudonymised datasets transmitted for linkage onto OpenSAFELY are encrypted; access to the platform is via a virtual private network (VPN) connection, restricted to a small group of researchers; the researchers hold contracts with NHS England and only access the platform to initiate database queries and statistical models; all database activity is logged; only aggregate statistical outputs leave the platform environment following best practice for anonymisation of results such as statistical disclosure control for low cell counts.[2]

The OpenSAFELY research platform adheres to the obligations of the UK General Data Protection Regulation (GDPR) and the Data Protection Act 2018. In March 2020, the Secretary of State for Health and Social Care used powers under the UK Health Service (Control of Patient Information) Regulations 2002 (COPI) to require organisations to process confidential patient information for the purposes of protecting public health, providing healthcare services to the public and monitoring and managing the COVID-19 outbreak and incidents of exposure; this sets aside the requirement for patient consent.[3] This was extended in July 2022 for the NHS England OpenSAFELY COVID-19 research platform.[4] In some cases of data sharing, the common law duty of confidence is met using, for example, patient consent or support from the Health Research Authority Confidentiality Advisory Group.[5]

Taken together, these provide the legal bases to link patient datasets on the OpenSAFELY platform. GP practices, from which the primary care data are obtained, are required to share relevant health information to support the public health response to the pandemic, and have been informed of the OpenSAFELY analytics platform.

This study was classified as service evaluation. This study was supported by Professor Martin McKee (Honorary Consultant at UCLH NHS Foundation Trust) as senior sponsor, and approved by the University of Liverpool's Research Ethics Board [reference 10634].

References:

For peer review only - http://bmjopen.bmj.com/site/about/guidelines.xhtml

1 Data Security and Protection Toolkit - NHS Digital. NHS Digital. https://digital.nhs.uk/data-and-information/looking-after-information/data-security-and-information-governance/data-security-and-protection-toolkit (accessed 30 Apr 2020).

2 ISB1523: Anonymisation Standard for Publishing Health and Social Care Data - NHS Digital. NHS Digital. https://digital.nhs.uk/data-and-information/information-standards/information-standards-and-data-collections-including-extractions/publications-and-notifications/standards-and-collections/isb1523-anonymisation-standard-for-publishing-health-and-social-care-data (accessed 30 Apr 2020).

3 Secretary of State for Health and Social Care - UK Government. Coronavirus (COVID-19): notification to organisations to share information. 2020. https://web.archive.org/web/20200421171727/https://www.gov.uk/government/publications/coronavirus-covid-19-notification-of-data-controllers-to-share-information

4 Secretary of State for Health and Social Care - UK Government. Coronavirus (COVID-19): notification to organisations to share information. 2022. https://www.gov.uk/government/publications/coronavirus-covid-19-notification-to-organisations-to-share-information/coronavirus-covid-19-notice-under-regulation-34-of-the-health-service-control-of-patient-information-regulations-2002

5 Confidentiality Advisory Group. Health Research Authority. https://www.hra.nhs.uk/about-us/committees-and-services/confidentiality-advisory-group/

Data access
Access to the underlying identifiable and potentially re-identifiable pseudonymised electronic health record data is tightly governed by various legislative and regulatory frameworks, and restricted by best practice. The data in OpenSAFELY is drawn from General Practice data across England where EMIS and TPP are the data processors.

EMIS and TPP developers initiate an automated process to create pseudonymised records in the core OpenSAFELY database, which are copies of key structured data tables in the identifiable records. These pseudonymised records are linked onto key external data resources that have also been pseudonymised via SHA-512 one-way hashing of NHS numbers using a shared salt. Bennett Institute for Applied Data Science developers and PIs holding contracts with NHS England have access to the OpenSAFELY pseudonymised data tables as needed to develop the OpenSAFELY tools.

These tools in turn enable researchers with OpenSAFELY data access agreements to write and execute code for data management and data analysis without direct access to the underlying raw pseudonymised patient data, and to review the outputs of this code. All code for the full data management pipeline—from raw data to completed results for this analysis—and for the OpenSAFELY platform as a whole is available for review at github.com/OpenSAFELY.

The data management and analysis code for this paper was led by Dr Mark Green and contributed to by all named authors.

For peer review only - http://bmjopen.bmj.com/site/about/guidelines.xhtml

For peer review only

For peer review only - http://bmjopen.bmj.com/site/about/guidelines.xhtml

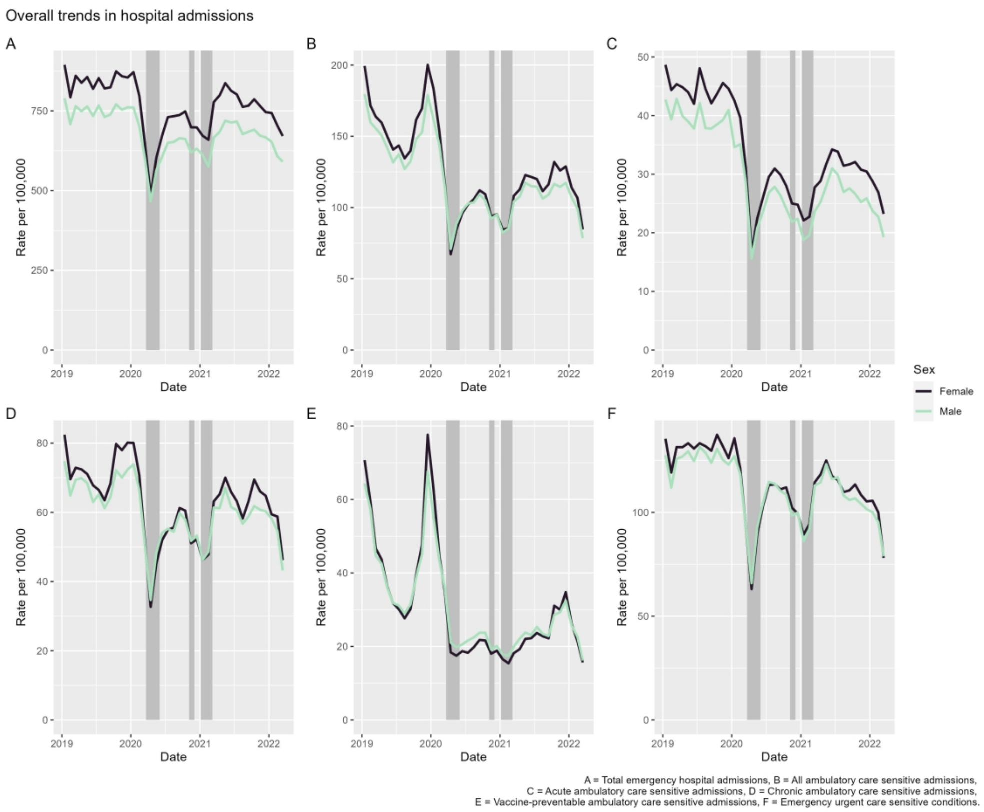

Figure 1: Crude hospital admission rates (per 100,000 population) by sex for measures of avoidable hospitalisations. Note: shaded periods represent national lockdowns.

1272x1030mm (72 x 72 DPI)

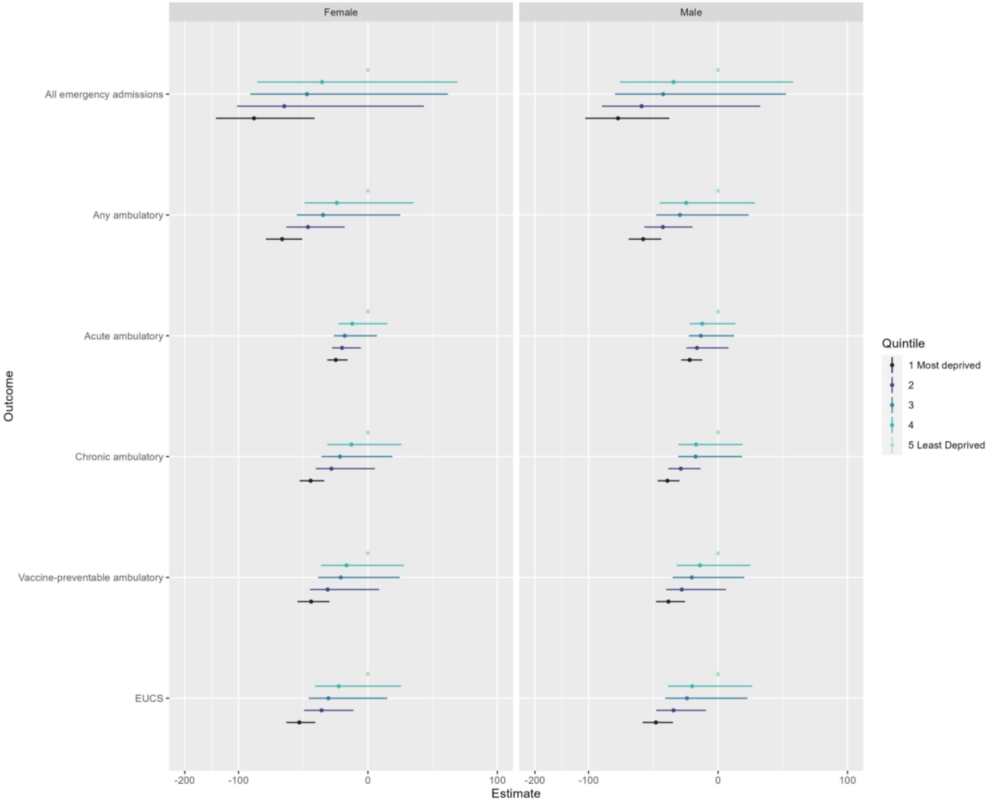

Figure 2: Estimated interaction effects (with 95% confidence intervals) between start of the pandemic and deprivation quintile from a series of difference-in-differences regression models for six outcome variables. Note: EUCS = Emergency Urgent Care Sensitive admissions. Ambulatory = ambulatory care sensitive admissions. Least deprived quintile was selected as the reference group.

1272x1030mm (72 x 72 DPI)

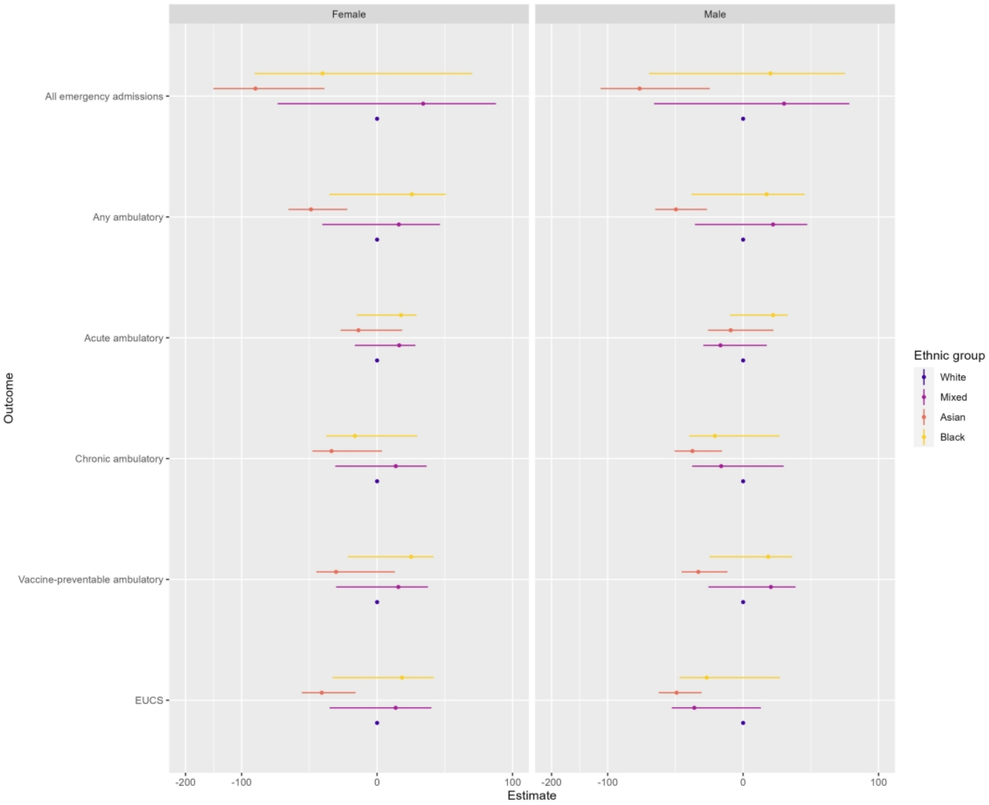

Figure 3: Estimated interaction effects (with 95% confidence intervals) between start of the pandemic and ethnic group from a series of difference-in-differences regression models for six outcome variables. Note: EUCS = Emergency Urgent Care Sensitive. Ambulatory = ambulatory care sensitive conditions. White ethnicity was selected as the reference group.

1272x1030mm (72 x 72 DPI)

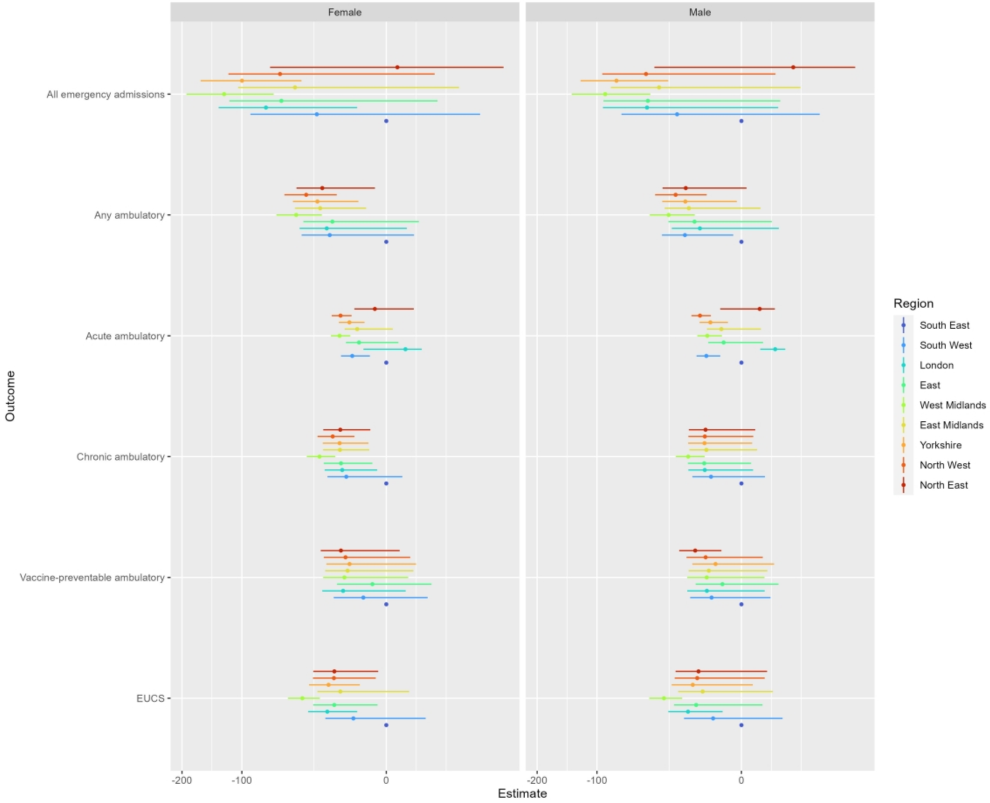

Figure 4: Estimated interaction effects (with 95% confidence intervals) between start of the pandemic and geographic office region from a series of difference-in-differences regression models for six outcome variables. Note: EUCS = Emergency Urgent Care Sensitive admissions. Ambulatory = ambulatory care sensitive admissions. South East region was selected as the reference group.

1272x1030mm (72 x 72 DPI)

Appendix

Table A: Missing data for covariates

Measure	Percent missing (%)
Index of Multiple Deprivation	1.68
Ethnicity	10.21
Government Office Region	0.05

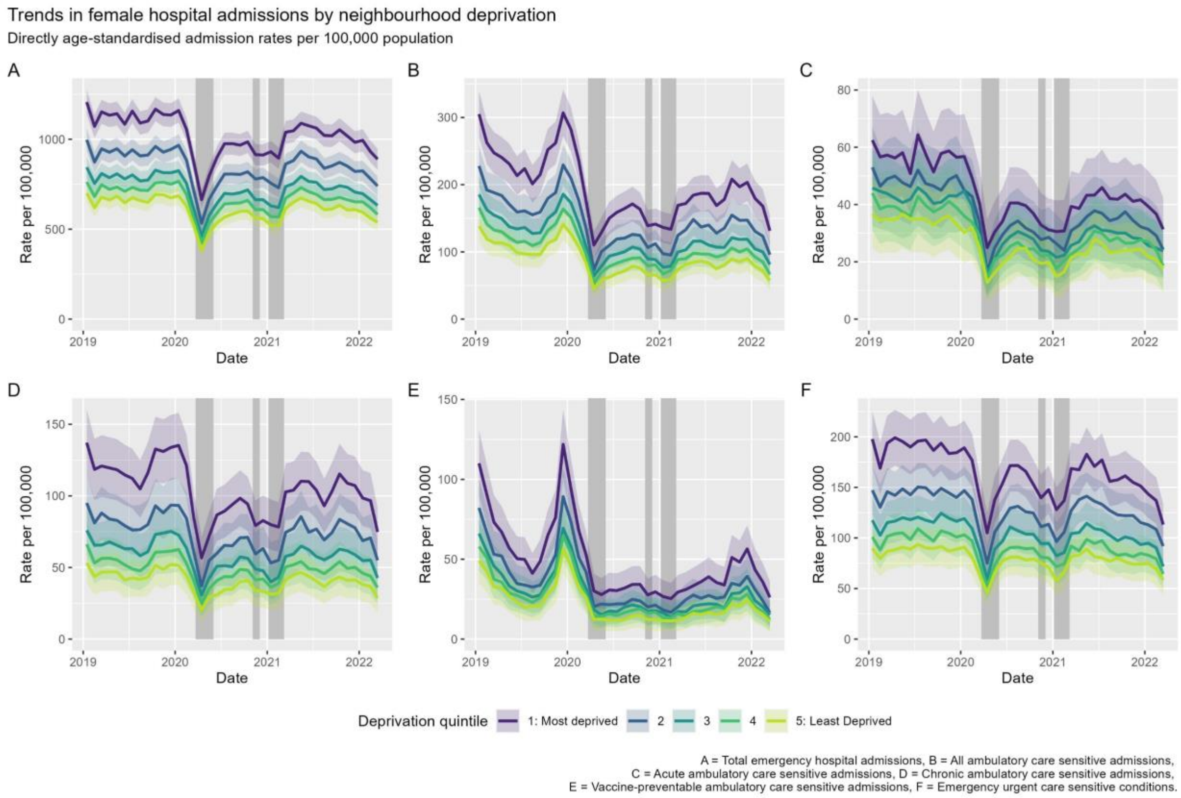

Figure A: Directly age-standardised admission rates for females by socioeconomic deprivation quintile. Note: shaded periods represent national lockdowns.

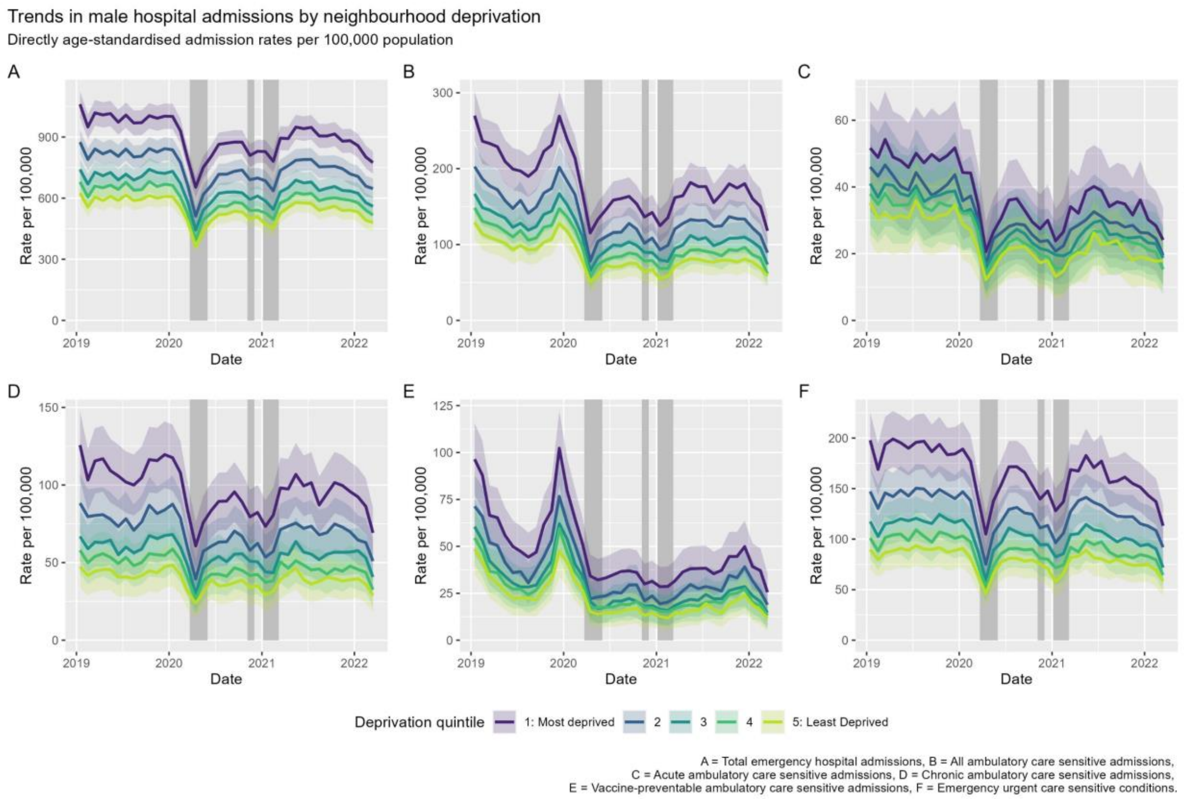

Figure B: Directly age-standardised admission rates for males by socioeconomic deprivation quintile. e: shaded periods represent national lockdowns.

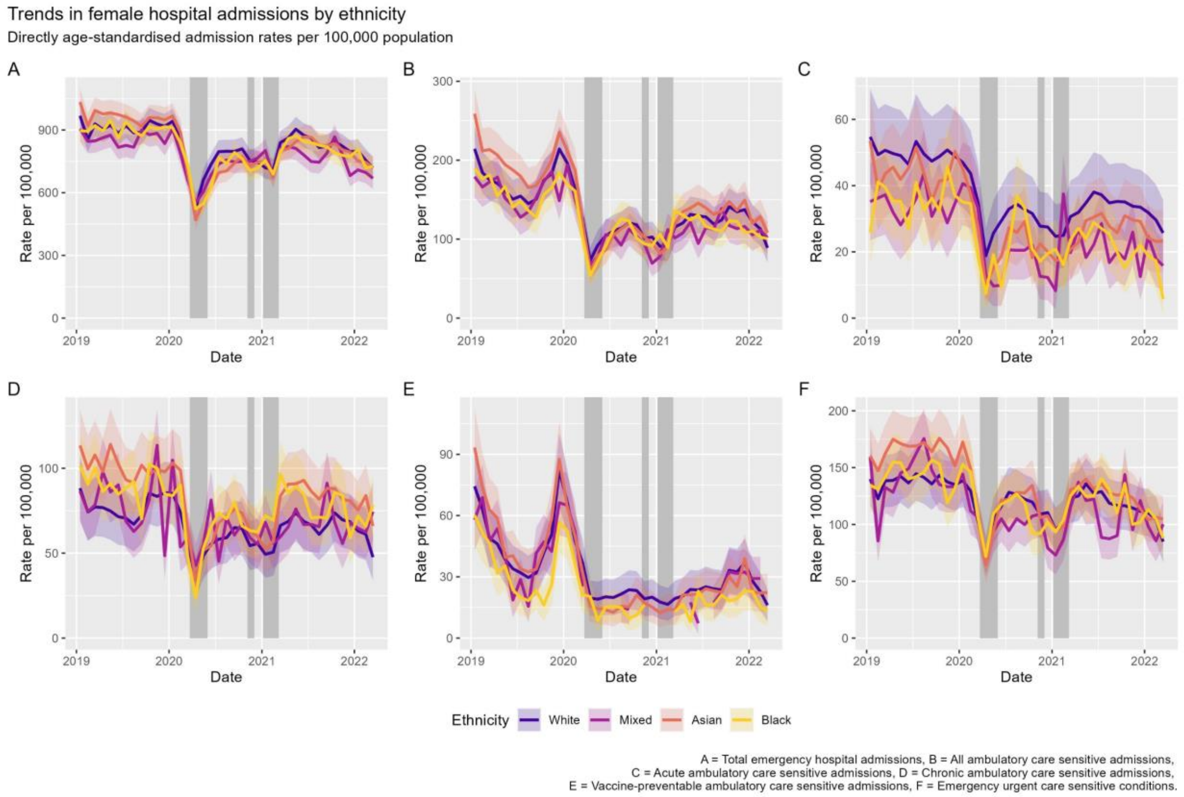

Figure C: Directly age-standardised admission rates for females by ethnicity. e: shaded periods represent national lockdowns.

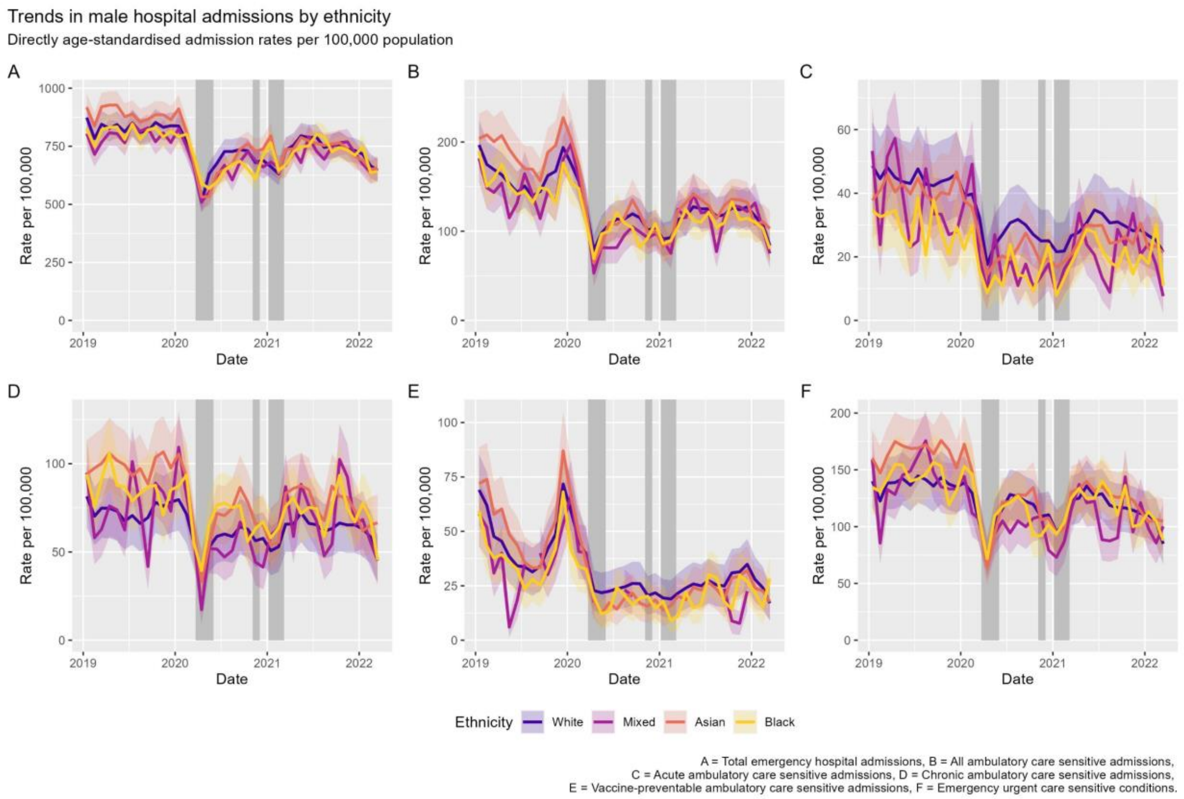

Figure D: Directly age-standardised admission rates for males by ethnicity. e: shaded periods represent national lockdowns.

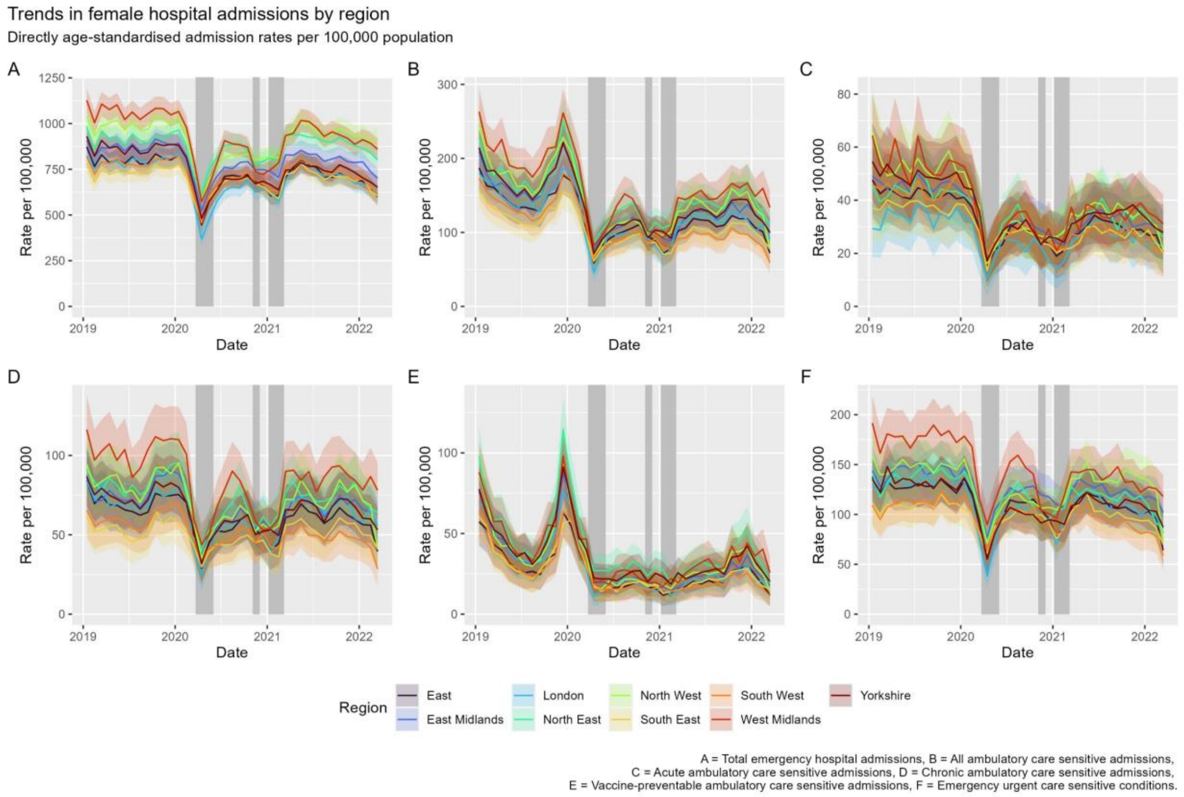

Figure E: Directly age-standardised admission rates for females by region. e: shaded periods represent national lockdowns.

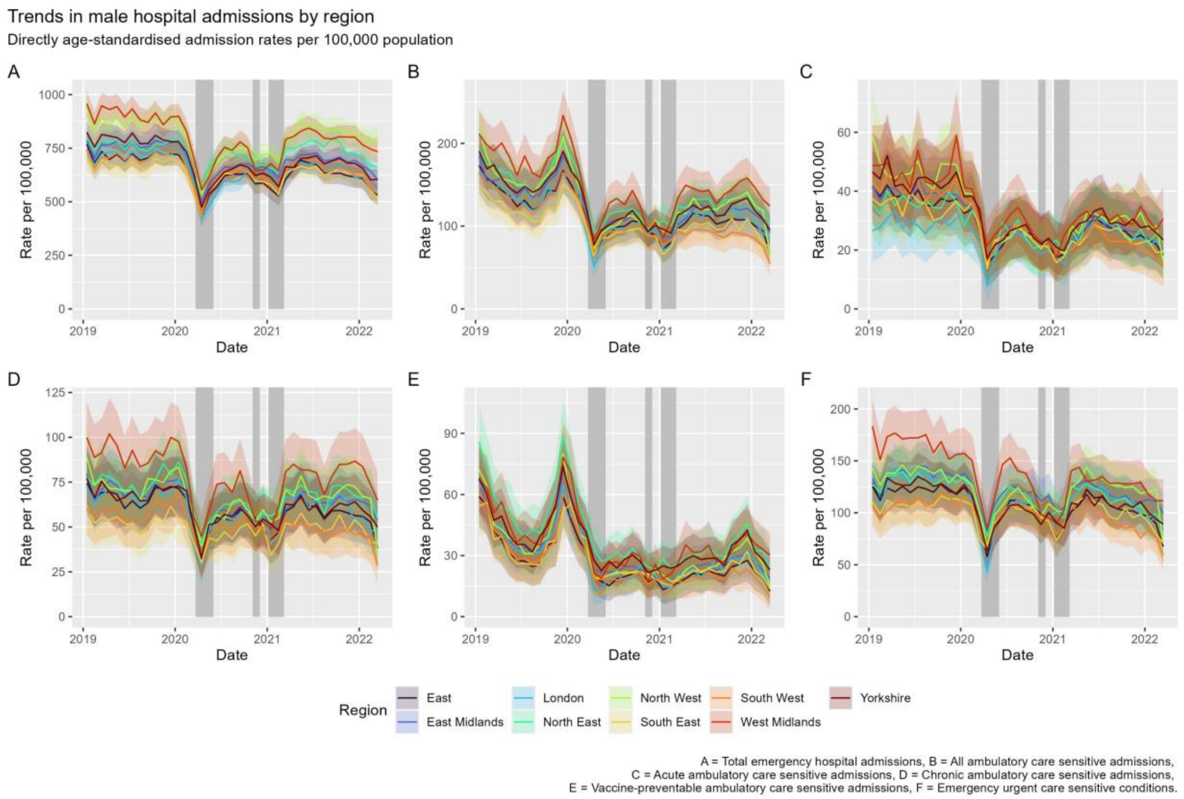

Figure F: Directly age-standardised admission rates for males by region. e: shaded periods represent national lockdowns.

For peer review only

STROBE 2007 (v4) checklist of items to be included in reports of observational studies in epidemiology*
Checklist for cohort, case-control, and cross-sectional studies (combined)

Section/Topic	Item #	Recommendation	Reported on page #
**Title and abstract**	1	(*a*) Indicate the study's design with a commonly used term in the title or the abstract	1
		(*b*) Provide in the abstract an informative and balanced summary of what was done and what was found	2
**Introduction**			
Background/rationale	2	Explain the scientific background and rationale for the investigation being reported	3
Objectives	3	State specific objectives, including any pre-specified hypotheses	4
**Methods**			
Study design	4	Present key elements of study design early in the paper	4
Setting	5	Describe the setting, locations, and relevant dates, including periods of recruitment, exposure, follow-up, and data collection	4
Participants	6	(*a*) *Cohort study*—Give the eligibility criteria, and the sources and methods of selection of participants. Describe methods of follow-up<br>*Case-control study*—Give the eligibility criteria, and the sources and methods of case ascertainment and control selection. Give the rationale for the choice of cases and controls<br>*Cross-sectional study*—Give the eligibility criteria, and the sources and methods of selection of participants	4
		(*b*) *Cohort study*—For matched studies, give matching criteria and number of exposed and unexposed<br>*Case-control study*—For matched studies, give matching criteria and the number of controls per case	
Variables	7	Clearly define all outcomes, exposures, predictors, potential confounders, and effect modifiers. Give diagnostic criteria, if applicable	4-5
Data sources/ measurement	8*	For each variable of interest, give sources of data and details of methods of assessment (measurement). Describe comparability of assessment methods if there is more than one group	4-5
Bias	9	Describe any efforts to address potential sources of bias	5
Study size	10	Explain how the study size was arrived at	4
Quantitative variables	11	Explain how quantitative variables were handled in the analyses. If applicable, describe which groupings were chosen and why	4-5
Statistical methods	12	(*a*) Describe all statistical methods, including those used to control for confounding	5
		(*b*) Describe any methods used to examine subgroups and interactions	5
		(*c*) Explain how missing data were addressed	5
		(*d*) *Cohort study*—If applicable, explain how loss to follow-up was addressed<br>*Case-control study*—If applicable, explain how matching of cases and controls was addressed	N/A

For peer review only

		Cross-sectional study—If applicable, describe analytical methods taking account of sampling strategy	
		(*e*) Describe any sensitivity analyses	N/A
**Results**			
Participants	13*	(a) Report numbers of individuals at each stage of study—eg numbers potentially eligible, examined for eligibility, confirmed eligible, included in the study, completing follow-up, and analysed	6
		(b) Give reasons for non-participation at each stage	N/A
		(c) Consider use of a flow diagram	N/A
Descriptive data	14*	(a) Give characteristics of study participants (eg demographic, clinical, social) and information on exposures and potential confounders	6
		(b) Indicate number of participants with missing data for each variable of interest	6
		(c) *Cohort study*—Summarise follow-up time (eg, average and total amount)	N/A
Outcome data	15*	*Cohort study*—Report numbers of outcome events or summary measures over time	N/A
		Case-control study—Report numbers in each exposure category, or summary measures of exposure	N/A
		Cross-sectional study—Report numbers of outcome events or summary measures	6
Main results	16	(*a*) Give unadjusted estimates and, if applicable, confounder-adjusted estimates and their precision (eg, 95% confidence interval). Make clear which confounders were adjusted for and why they were included	6-8
		(*b*) Report category boundaries when continuous variables were categorized	N/A
		(*c*) If relevant, consider translating estimates of relative risk into absolute risk for a meaningful time period	N/A
Other analyses	17	Report other analyses done—eg analyses of subgroups and interactions, and sensitivity analyses	6-8
**Discussion**			
Key results	18	Summarise key results with reference to study objectives	8
Limitations	19	Discuss limitations of the study, taking into account sources of potential bias or imprecision. Discuss both direction and magnitude of any potential bias	10-11
Interpretation	20	Give a cautious overall interpretation of results considering objectives, limitations, multiplicity of analyses, results from similar studies, and other relevant evidence	8-10
Generalisability	21	Discuss the generalisability (external validity) of the study results	10-11
**Other information**			
Funding	22	Give the source of funding and the role of the funders for the present study and, if applicable, for the original study on which the present article is based	14

*Give information separately for cases and controls in case-control studies and, if applicable, for exposed and unexposed groups in cohort and cross-sectional studies.

Note: An Explanation and Elaboration article discusses each checklist item and gives methodological background and published examples of transparent reporting. The STROBE checklist is best used in conjunction with this article (freely available on the Web sites of PLoS Medicine at http://www.plosmedicine.org/, Annals of Internal Medicine at http://www.annals.org/, and Epidemiology at http://www.epidem.com/). Information on the STROBE Initiative is available at www.strobe-statement.org.

For peer review only
